# A Further Inquiry Concerning Constitutional Irritation, and the Pathology of the Nervous System

**Published:** 1835-10-01

**Authors:** 


### Medico-Chirurgical Review,

No. XLVI.

[No. 6 of a Decennial Series.]

JULY 1, TO OCTOBER 1, 1835.

### A FURTHER INQUIRY CONCERNING CONSTITUTIONAL IRRITATION, AND THE PATHOLOGY OF THE NERVOUS SYSTEM. By *Benjamin Travers*, F.R.S. Senior Surgeon to St. Thomas's Hospital, &c. &c. Octavo, pp. 444. Longman's, London, 1835.

OUR readers may remember that, in the year 1827, Mr. Travers published the first part of a work, entitled, "An Inquiry concerning that Disturbed State of the Vital Functions usually denominated Constitutional Irritation." The interval between the appearance of that and of the present Part has been considerable, but Mr. Travers does not deem it necessary to vindicate himself from the imputation of having pursued an idle speculation, because he has no novelties to submit to the notice of the public. His object may be gleaned from the following brief apology.

"I request of my reader to bear in mind that it professes to be only an 'Inquiry,' seeking and supplying illustration from facts, and reasonings thereupon, not aspiring to guide his views so much as to unfold my own; and thus to indicate the direction in which, as it appears to me, his also may be usefully applied. For the rest, I bespeak his patience and his candour;—the first, that he may duly appreciate the extent and importance of the subject; the second, that he may judge of the manner in which it is treated with forbearance, and lenity to its admitted defects.—' Res enim ardua vetustis novitatem dare, novis autoritatem, obscuris lucem, dubiis fidem; omnibus Naturam, et Naturæ sua omnia."—*Preface*, viii.

The work before us consists of two divisions. The first is devoted to the consideration of Irritation of various kinds—the second to the Nervous System and its Pathology.

The first contains six chapters. The two first are occupied with the subject of "reflected irritation." In the third we are informed of the connexion between direct and reflected irritation. In the fourth we are made acquainted with the local changes of structure not essentially inflammatory, and of local inflammations supervening on or connected with constitutional diseases. In the fifth, we are presented with constitutional inflammations as examples of reflected irritation. And, finally, the sixth is a supplement to the preceding five.

To this first division we shall now proceed, and endeavour to exhibit a connected view of the facts and the opinions of our author. In this attempt we perceive and must admit the existence of a difficulty. Our author's ideas are profound, and his language is at times obscure. The subject, Irritation, is one more talked about than understood, or indeed intelligible, and though we must in practice admit its existence, in reasoning we are scarcely able to explain its nature. This reflection may account for what might otherwise appear confused in the language or the thoughts of Mr. Travers. What remarks we have to make will chiefly spring from a consideration of individual facts or opinions. We shall therefore advance, without more delay, to an analytical examination of the work.

The first chapter opens with a recapitulation of the principal objects of the former volume. His principal aim was to exhibit the occurrence, the phenomena, and the results of that state termed by surgeons Constitutional Irritation.

"I drew," he observes, "a distinction between irritation and inflammation in a local sense, and irritation and fever in a general sense; shewing at the same time, that though the combination in both senses was frequent, it was far from invariable; and that, upon the whole, the most formidable, because the least controllable forms of constitutional irritation existed in the absence of inflammation and fever.

The nervous system I shewed to be the source of these morbid actions, and pointed out the direct agency of some causes of excitement, and the indirect agency of others, and thence established the distinction between direct and reflected irritation. It was my object, in the former work, to delineate the direct, and in the present I shall consider the reflected irritation. It is essential to premise, that I confine my view to such diseases as have sooner or later external and local demonstration, and are therefore strictly in the province of surgery; although such demonstration may be trivial in appearance, or in point of time separated by a considerable interval from the constitutional disturbance, whether anterior or subsequent to it. To recapitulate.—

'Constitutional irritation I consider to be of two kinds, direct and reflected, by which arbitrary distinction I mean to imply, that the first is wholly and immediately derived from the part, commences and is identified with the local mischief, and the constitution has no share in its production. The second, on the contrary, originates in a peculiar morbid state of the constitution, to which the injury or inflammation has given birth, or it may be, previously existing. The first is truly symptomatic; never originating spontaneously, and being immediately induced by the local irritation, is capable of being essentially mitigated or arrested by its removal. The second is occasionally idiopathic, and being as often the cause as the effect of the local action, is less influenced by local treatment. In the first, the local changes are depending on local causes, in the second they depend on constitutional causes. Cases are of no uncommon occurrence, in which after an interval the reflected supervenes upon the direct irritation, or the direct is superadded to the reflected, already established, which may therefore be regarded as examples of mixed irritation, the part and the constitution acting and reacting alternately upon each other.'" 3.

With this recapitulation, which constitutes a sort of formal introduction to the reader, and must establish an acquaintance between him and the author, we feel ourselves at home with both, and may proceed, without restraint, to facilitate their mutual intercourse.

Mr. Travers occupies the remainder of this chapter with a sketch of the influence of constitution and habits upon local disease. The sketch is a just one, more just indeed than novel, and requires no particular notice at our hands. The experience of mankind has always recognized and must ever own the operation of both these agencies. The conclusions of our author may be briefly summed up. If called upon to mention the most powerful predisponents to disease, he would say, of the time of life, youth or age; of temperaments, the scrofulous; of habits of life, those of poverty in the aggregate; of moral causes, anxiety and the depressing passions; of external and adventitious circumstances, the abuse of liquor, the operation of cold, and the action of mercury. To these conclusions we see no objection, indeed we suppose they would be generally admitted, and are generally believed.

The subjects of the second chapter are—Illustrations of Reflected Irritation—Pre-existing Organic Disease—Metastasis—Diseased Actions set up in other parts by Injuries and Inflammations—and Cases illustrative of all.

1. *Pre-existing Organic Disease.* The gist of Mr. Travers' remarks upon this head may be said to be, that organic changes silently creep on, and that few attain lengthened age without them, whilst few also die from disease or accident after the middle period of life, in whom dissection does not disclose some palpable evidences of organic change not previously known, or at best only vaguely surmised to exist.

We are inclined to think that Mr. Travers attaches much importance to the more minute alterations of structure, that occur as life advances—and that he leans to the opinion that the result of operations is greatly influenced by what would seem to be slight organic changes. We suppose, there are few persons who would broadly deny the general position, that the slightest departure from a perfectly sound state of any organ, must constitute pro tanto a departure from good health, and constitutional vigour. Yet the application of this rule is difficult, and many disturbing circumstances conspire to diminish its usefulness as a practical standard of truth. Mr. Travers presents some cases, calculated to support his opinions upon this point. He has notes of others in which small operations have proved fatal in a week or ten days, in a manner quite inexplicable but upon the presumption of a pre-existing morbid diathesis, or an actual morbid change; when the friends of the parties, in their vexation at so unexpected a result, have immediately removed the body, and obstinately refused inspection. A morbid condition of the lungs or liver, and effusion into the chest or upon the brain, have generally been indicated in such instances. Yet he candidly adds that, in other cases where examination has been obtained, no actual change has been detected beyond a gorged condition of the blood-vessels of these organs, partial depositions from their extremities, opacities, and old adhesions of the lining membranes, extreme flaccidity of the muscular fibre, &c. It must be owned that in many instances patients die after injuries and operations, when no previous organic changes can be found to help the pathologist out of his difficulties, or assist him in his conjectures. It is well when science makes all clear, and when it fails, as it often does, to tell us precisely the why and wherefore, it induces us to search more deeply, and to strive more manfully to observe, to compare, and to discover.

Amongst other cases, Mr. Travers cites the following.

*Case.* A stout brew-house porter, æt. 54, had the fluid of a hydrocele drawn off by a lancet-puncture on the 1st of April, 1834. On the 8th, he felt chilly and feverish, and a slight erythematous blush was observed over the now again tense scrotum and groin of the same side. The scrotum was freely incised, and an abscess discharged. The cellular membrane was in a state of slough. On the 18th the skin had become yellow; on the 21st, he was attacked with purging; on the 22d, he had a rigor, and the wound was in a state of gangrene; on the 24th, he died. This man had almost lived on porter.

On examination, the tunica vaginalis was sloughy, but the affection of the groin was confined to the skin: the testis and chord sound, and a small hydrocele of the latter. There was a gorged condition of all the viscera. The liver was brittle and granulated. Some chronic adhesions were found in the chest.

Such cases as these are common in the London hospitals. A man who has been accustomed to drink large quantities of gin or porter, and to eat perhaps much animal food, receives a compound fracture. He is placed in bed, put suddenly on low diet, and deprived of the stimulants he has been accustomed to. Such a man gets delirium tremens, or low diffuse inflammation of the cellular texture of the limb. He dies, and on dissection, the liver is found to present what is called the "nutmeg" appearance, or perhaps no obvious visceral alteration can be found. Mr. Travers selecting the condition of the liver, or, it may be, some old adhesions in the chest, or a few scattered tubercles in the lung, as evidence of pre-existing organic disease, sees in this sufficient cause why the patient dies, from a slight injury or a trivial operation.

Yet we doubt if his opinion is that of the generality of surgeons. They are rather disposed to look upon the patient as one who has been used to an unnatural stimulus, and who cannot support the sudden and continued abstraction of it. They watch with anxiety for the symptoms that denote the advent of low fever, and they cautiously refrain from keeping the individual too long on a debilitating regimen. We recollect the period, it is not many years ago, when traumatic delirium, and diffuse inflammation of the cellular tissue, and the low forms of erysipelas were much more frequent after injuries and operations in a large London hospital than they are at present. Then attention had scarcely been directed to the necessity of stimulating patients who had habitually been stimulated. Now such accidents are much less common, because the depletion of persons of this description is carefully avoided. Yet probably as many "nutmeg livers" are produced by gin as formerly, and perhaps it may be found that an equal quantity of "pre-existing organic disease," is not attended with a corresponding quantum of mortality.

For our own parts, we will not attempt to maintain or to controvert the position, that a trivial amount of cognizable organic alteration is sufficient to account for the fatal results, occasionally, and but occasionally, noticed after operations usually unimportant. The discovery of these changes may seem to offer a satisfactory explanation of the circumstances, but unfortunately we are met with the obvious difficulty, that changes much greater seem to be productive of no serious consequences.

We think we may leave this question to those who consider themselves capable of sifting or of solving it, and may pass to the second "Illustration of reflected irritation," offered by our author.

2. *Metastasis.* Mr. Travers seems to think that the principle of metastasis is, in effect, the same as that of sympathy. This may be thought an attempt to illustrate the obscurum by the obscurius, and we conceive that an example or two of the fact is, in the present condition of our knowledge, worth, perhaps, more than a volume of discussion.

"Of several instances in which I have witnessed the fatal operation of this principle, I should mention as among the most marked, serous apoplexy from retention of urine and the sudden reduction of swollen testes; palsy of the retina from the sudden retrocession of a skin rash, from a tumor obstructing the bowels, the irritation of worms, engorgement of the liver, and suppressed catamenia; and erythema, erysipelas, and even diffused gangrenous inflammation from the healing of wounds upon distant parts. But the case most strongly impressed upon my mind happened many years ago, in which I encouraged one of my dressers to a trial of skill in accomplishing the healing of a very extensive and ancient ulcer of the leg, within a given time. Almost immediately upon its complete cicatrization, a diffused swelling arose in the pectoral region of the same side, the parietes took on extensive gangrenous inflammation, and the patient died in less than a week." 20.

Mr. Travers relates four cases. They resemble each other so very closely, that one is a specimen of all. Whether that will be thought a fair example of metastasis is another thing.

*Case.* A sailor, lately returned from the West Indies, of sallow complexion, but by his own report, good health, with regular bowels, unimpaired appetite, clean tongue, and steady pulse, had a large deep sloughy ulcer on the outer side of the right foot, and another on the left leg; they were of two months's standing, and had been much neglected on ship-board.—Bark, and a small allowance of wine and porter. A stale beer poultice to each sore. On 8th of August, 1818, the sloughs had disappeared from the sores, and healthy granulations had sprung up. On the 15th, there was some enlargement of the abdomen. On the 12th of September, the sores were nearly healed, and ten quarts of limpid fluid were drawn off by tapping the abdomen. The fluid re-accumulated, and, on the 15th of October was again drawn off. The sores were healed. For the third time the abdomen filled, and, on the 20th of November, the patient died.

For our own parts, we must own that the evidence in favour of metastasis is rather weak. The patient, just returned from the West Indies, was in a state which rendered dropsy very likely to ensue. That he did get ascites, when ulcers that existed on the leg were improving, is certainly not conclusive of the occurrence of metastasis. Yet this case is quite as satisfactory as the rest. We would not wish to be misunderstood. We do not question the occurrence of metastasis. We have seen it more than once, and its reality is, we think, as completely established as that of any principle of action in the animal economy. But Mr. Travers' instances are unfortunate; a critic might reasonably object to them.

The third illustration of reflected irritation is—

3. *Diseased Actions set up in other Parts, by Injuries and Operations.*

"In the works of various authors and collectors of cases, will be found examples of the post-mortem discovery of a morbid state of other and distant parts of the body, bearing every character of recent formation, and of the existence of which, prior to the injury in one case, and the external disease in another, there was neither sign nor ground for conjecture. These have sometimes been of a directly fatal character, as acute inflammation and effusion into the cavities. Sometimes, and more frequently, they have been of a chronic kind, opposing insurmountable obstacles to recovery by change of structure in vital organs, as tubercles in the lungs, liver, &c.; in other cases, they have operated by their diffusedness rather than their nature to oppress the powers of life, and thus aggravate the malady, as extensive inflammatory actions upon the skin and cellular membrane. Lastly, internal organs have assumed a character of disease in a more advanced stage, or a more important position, similar to the first or external malady, whether scrofulous, or belonging to the morbid poisons, as the scirrhous and medullary cancer, lues, &c." 23.

Mr. Travers next refers, and rather fully, to the paper of the late Mr. Rose, on the subject of secondary inflammations after injuries and operations. To this subject, we have probably directed quite as much attention as our author, and our readers have been made acquainted with the facts and opinions of later inquirers—Arnott, Velpeau, and others. In the midst of this notice of Mr. Rose's paper, Mr. Travers introduces the following case.

*Case.* " A young lady, whose illness occupied a period of two years, was treated with great attention by several of the most eminent of the profession, in the departments of physic, surgery, and midwifery. Opinions were divided on the point, whether the brain was affected in its organization, or only in its function; the symptoms being, circumscribed fixed pain over the left limb of the lambdoid suture increased by pressure; pain in the extremities, and great loss of muscular power, nearly amounting to hemiplegia of the same side. Signs of great depression of the whole nervous system were alarmingly indicated, and the disturbance of the external senses and mental faculties was such, as accompanied with obstinate obstruction and continued wasting, left no one in doubt as to the seat and result of the malady; although an uneasiness approaching to pain at the lower margin of the chest on the same side and dyspnœa, supposed spasmodic, called for the occasional application of three or four leeches, which gave temporary relief to these symptoms. The prostration and distress from motion of any kind were excessive. Was it organic disease of the brain, or aggravated hysteria?

*Examination.* The brain and its membranes, and all the viscera were healthy, with the exception of the lungs, which were filled with tubercles in various states of progress. No apprehension of this fact had been entertained through the whole progress of the malady, so completely had the metaphysical or functional masked the physical or organic condition." 25.

What this case has to do with the occurrence of secondary inflammations after operations and injuries, we profess ourselves quite unable to conceive. The above was an instance of tubercular phthisis, undiscovered during life, and it seems to us to have been little else. In fact, Mr. Travers seems to mix up many various actions and affections, and to view them, we think without advantage, under one general heading. However that may be, he divides these secondary actions into contiguous and remote. The continuous sympathy of parts is illustrated by ordinary examples, being a direct oftener than a reflected irritation. The affection commonly called erysipelas, in which the cellular membrane of the entire arm becomes progressively the seat of inflammation, from an injury to the tip of the finger, presents the most apposite and familiar instance. He offers, also, instances of contiguous inflammatory action. Take one. A deeply situated steatomatous tumor was extirpated from the axilla. The man died on the eighth day from acute pleurisy of the same side. No suspicion was entertained of the inflammation of the thorax, although the right lung was covered with patches of recent lymph, and a copious effusion had taken place in the cavity. In reference to this case, we think it most probable that the contiguity, or otherwise, of the pleura was really a matter of no consequence. Another person has the great toe amputated, and he, too, dies of recent pleurisy. Are the cases dissimilar? Only, it appears to us, in the accidental circumstance, that the great toe is farther from the chest than the axilla is. The affections are parallel, and divisions of name, and of name only, are, perhaps, of dubious advantage.

Mr. Travers relates several cases of remote "inflammatory action"—pericarditis, for instance, after a suppurating scalp-wound—peritonitis, after amputation of a leg for fungoid tumor—phthisis, after ulceration of the cartilages of the knee-joint, and so on. That all are instances of inflammatory action, supervening in an organ remote from that originally implicated, can neither be doubted nor disputed. Yet, although nosologically classed together, there are many and important differences between the secondary symptoms of syphilis, the secondary deposites after an operation or an injury, and the phthisis that follows a chronic articular disease. This instance serves, like others, to shew that works of this description are fit only for those, whose information and experience enable them to take general doctrines and speculative opinions, for no more than they are fairly worth.

We have now arrived at the third chapter, which is headed.

*Of the Connexion between Direct and Reflected Irritation—their Reciprocity and Mixed Operation—the System re-acting on the Part, and the Part on the System.*

The object of our author, in this chapter, is beset with no inconsiderable difficulties. Irritation, or, at least, the explanation of it, is not the most comprehensible of actions, and the mode of connexion between its forms, direct or reflected, may, perhaps, be more readily imagined than described. An author of talent, conversant with logical and metaphysical distinctions, frequently labours under the misfortune of addressing himself to readers, to whom metaphysics are equally unintelligible and uninteresting. Utilitarians in reasoning, as in fact, the immediate cui bono is their ready reply to an ingenious tissue of philosophic speculations. That class of readers will, we fear, feel some disappointment at the mode in which Mr. Travers treats of the connexion between the kinds of irritation we have mentioned. We shall endeavour to state his opinions clearly, though we fear we labour under the disadvantage of not exactly understanding them.

We are disposed to commence with a passage with which the chapter terminates. Its gist is an eulogium on the genius of Mr. Abernethy, and the value of induction. Yet we doubt if our author's view of induction does not trench a little on the realms of speculation, and we are not sure that the extension he accords to his, would submit to the more rigid trammels of the Baconian rules.

"The phenomena of reflected, *i. e.* reciprocal irritation, require to be more carefully studied than they have hitherto been by those who consider the profession of surgery a branch of science, and not a purely mechanical art. It is only of late years that the pathology of surgery has been cultivated, and for this we are mainly indebted to the genius of Mr. Abernethy, who possessed a mind of too philosophical a cast to be satisfied with the bare observation of facts, leaving their analysis and the relation between effects and their causes to more curious enquirers. There is a restlessness about a mind endowed with the power of investigation by induction, to trace the connexion between phenomena which are obvious to the senses, and those which, though less apparent, are not less real. The share which the constitution takes in diseases falling strictly within the province of the surgeon is so important, and exercises so vast an influence over the event, that I cannot but feel more surprised at the credit which our predecessors obtained, in neglect of such enquiries, than at the advances which the last twenty years have witnessed in the scientific improvement of surgery. A more perfect physiology is necessary, I admit, to explain much of what we see every day—but next to the consciousness of this deficiency, I believe that a habit of reflecting carefully upon morbid phenomena to the extent warranted by such data as we possess, will prove the strongest incentive, as well as the most efficient instrument, towards the improvement of physiology. The evils to be apprehended from applying the faculties of our minds to this object are first, an over-minuteness in our investigations, to the frittering away or oversight of master facts, or, on the other hand, a too rapid adoption of general principles and conclusions from insufficient particulars. The first is an error common to the humbler class of intellect, though not always that of least pretension—the latter is the rock upon which natural quicksightedness and ambitious enthusiasm, wanting the discipline of patient and laborious and truthful habits, so often make shipwreck of reputation." 63.

With this understanding of our author's general views, we may pass to his explanation of the connexion between direct and reflected irritation.

Sympathy he says, comprises the whole mystery of irritation. This, it must be admitted, is driving out devils by Beelzebub, the prince of the devils. Were it put to the majority of surgeons, to pronounce which they understand most clearly—sympathy or irritation, we are inclined to think that the majority would be puzzled what answer to return. He proceeds to observe, that if an irritation, strictly local in its origin, produce a certain degree of reaction, the determination to the nerves and bloodvessels of the part presents a series of phenomena, constituting inflammation. If the action begins and terminates in the part, without occasioning the consciousness or the sympathy of the part, the inflammation may be denominated local; but if the action of the part be such as to excite the sympathy of the system, a fever follows. This fever, arguing the absence of "a torpid, irresponsive; and unhealthy system," must be considered as serving a salutary purpose, the re-action being indispensable to the recovery of the part. This is, in fact, a version of the old maxim—the vis medicatrix Naturæ.

"When the injury, from its nature or its seat, is one of extraordinary severity, the constitution is affected to great febrile excitement or depression, as acute inflammatory or typhoid fever, but still presenting the type of fever. In extreme cases, however, as those of complicated injury, although neither involving vital organs nor the loss of blood, it loses that character altogether, and the system is permanently sunk beyond recovery, or is stunned and prostrated for a time, and recovers only to display new and anomalous modes of excitement. These, it will be remembered, are the two forms of direct irritation which I have formerly described—'prostration without reaction,' and 'prostration with excessive reaction,' as taking the place of fever in cases of grave local mischief, whether occasioned by violence or inflammation." 43.

The ordinary case of reflected irritation, says our able author, is that in which the system operates upon the part, or upon any remote part prejudicially, or in a manner destructive to its own safety. This is shewn in the supervention of erysipelas after injuries—in the insidious inflammation of remote parts after the same—and in "the preternatural actions of the nervous system, as trismus and tetanus." We feel it impossible to substitute any expressions of our own for the passage we are now about to quote. It is intended as an explanation of the supervention of direct upon reflected irritation.

"It will be obvious to reflection, that the direct irritation ensuing upon injury or inflammation, tends, if continued, to the production of the state of constitution above described; as when the injury, which acts as an exciting cause, though soothed, is unredressed, and the system is, by the aid of time and circumstance, restored to seeming tranquillity, though still oppressed by a sense of inequality to sustain the demands made upon it for support and repair. In this critical position it continually happens, that the constitution of the robust, which is unused to confinement, and of the weak, which is scarce adequate to maintain health, betrays a condition of morbid irritability which impedes the process of recovery, and lapses, sooner or later, into a wasting hectic, generally producing pulmonary or other visceral deposit. In those chronic changes of structure which arise independent of any assignable local cause, some of which we denominate constitutional diseases, as, for example, scrofula, scirrhus, &c., a similar passive morbid state of the system is gradually established, which, if aroused by any suddenly altered condition of the part, frequently assumes a degree of activity destructive to life. The irritation of a change, abstractedly beneficial, as for example, the removal of a tumor, or of a limb which we have vainly attempted to save, or the discharge of a large abscess, annihilates the benefit which, in different circumstances, it would have afforded. Had not the powers of the constitution been already overstretched, they would have rallied on the occasion, as they have often done from a state of almost hopeless depression; but the shock incidental to the change of condition oversets it, so exquisitely delicate has the balance become by gradual adjustment between the weight imposed and the sustaining force. If fatal results are exceptions to general experience, it is because experience has impressed caution; but they occur; and it has appeared to me that a more distinct solution of the question, why they occur, than has yet been given, may render them still less frequent.

This, then, is the case of the direct superadded to the reflected irritation; so that the reciprocity of the two is evinced in almost all protracted cases of surgery, and indeed their protraction is generally depending upon it. In a case which goes healthily forward without interruption, the constitution and the part preserve their adjustment so equally, that irritation can scarcely be said to exist; and this is the case when, after the first alarm and resistance of the nervous system is past, the circulation quiets down and lends all aid to the work of reparation. But when permanently unfavorable circumstances attend an injury or a disease, an ill-disposed habit or a cachexia is present, a previous organic change exists, or an interference takes place with the operations of nature, whether accidental, unavoidable, or ill-judged,—each of which causes comprehends a variety of conditions in detail,—the part and the constitution are alternate irritants, one of the other, and eventually co-operate for destruction." 46.

We fear that the rigid reasoner might cavil with the *induction* of much of the preceding passage. It is a plausible explanation of processes, more in the style of John Hunter than of that of severe modern pathology. It may be true, for example, that the system, while in a state of seeming tranquillity, is " still oppressed by a sense of inequality to sustain the demands made upon it for support and repair." This, we say, may be true ; but our author would find it difficult to prove this metaphorical consciousness and volition on the system's part. To state, also, as Mr. Travers states, that the constitution of a robust individual, unused to confinement, " betrays a condition of morbid irritability which impedes the process of recovery, and lapses, sooner or later, into a wasting hectic, generally producing pulmonary or other visceral deposit, is, when analysed, to say very little that is satisfactory or clear. In short, not to be arguing points which no argument will ever settle, we see, in these explanations, but little really explained; and we say this without any disrespect to Mr. Travers, who has chosen a subject that the present state of our knowledge, both of physics and of metaphysics, is incapable of placing within the pale of perfect comprehension and of demonstration. For our own parts, we must confess that our notions of direct and reflected irritation remain any thing but definite or positive.

Mr. Travers brings forward several cases to exemplify the superaddition of the direct to the already established reflected irritation, as best illustrating the reciprocity of the two, and shewing how natural and unavoidable circumstances may set it up, how slight and otherwise harmless, if not directly beneficial, treatment may act destructively through this medium.

*Case.* A gentleman, whose health was much impaired by a long continuance of that species of cachexia, rather absurdly termed pseudo-syphilis, accidentally struck his shin. The blow was followed by indolent inflammation, and the formation of matter beneath the periosteum. The latter was divided, and the patient was necessarily confined to bed. From the time of his taking to his room, this gentleman discovered an alarming impatience of confinement, and could with difficulty be kept at home. Though naturally cheerful, he became desponding, owing to his frequent relapses of indisposition, and being a man of a delicate and honorable mind, was not consoled by reflections on the predisposing cause of his malady. The wound assumed a sluggish, unhealthy character, its edges thickened, everted and painful, and a thin ichor flowing from it. The patient had neither rest nor appetite, and after three weeks' confinement to bed, a blush of inflammation appeared round the wound, and extended so rapidly, that in a day or two the entire limb, from the knee downward, was an œdematous, shapeless mass, fully twice the size of its fellow, and of a dusky red colour. While this change was taking place, the patient was seized with sudden delirium, so fierce as to require restraint, which was broken by intervals of the most obstinate and sullen silence, in which he appeared partially rational. After this state had endured three days and nights, he died exhausted.

In another case, a gentleman had extensive psoas abscess ; this was opened by an incision, an inch and a half long, and a large quantity of curdly matter discharged. A poultice was applied to the wound. For two days all went well ; but, on the night of the second day, the patient was attacked with nausea, vomiting, and rigor, and died in a state of collapse on the fourth day. On examination, an immense quantity of matter was found in the sacs of the abscesses, but no appearance of inflammation was discovered in the viscera or in their cavities.

In the case of a young man who had a small subcutaneous tumor, composed of condensed cellular and fatty substance above the inner condyle of the humerus, a blister was applied, and this was followed by diffuse inflammation of the arm. Two large abscesses ensued, the patient became delirious, and died three weeks after the application of the blister. The patient had been very anxious on account of the absence of his relations. Mr. Travers explains the case thus. The irritable state of the habit determined the severity of the local action on the small excitement of a crown-sized blister, and I have no doubt that the excision of the tumor would have been equally fatal. We question whether the pure spirit of induction permits such confident prognostications on contingencies.

Other cases are related, but they prove, or seem to prove, no more than those we have already quoted. If they do not throw light on the connexion between direct and reflected irritation, and render our author's ideas on this mysterious subject clear; we doubt if a multitude would do much more, and we therefore take up the succeeding Chapter.

*Of Local Changes of Structure not essentially Inflammatory. Of Local Inflammation supervening on Constitutional Diseases. Of Local Inflammation generating Constitutional Disease. Cachexia. Of Scrophula, Cancer, Syphilis, Cachectic Ulceration and Gangrene.*

Such is the multifarious heading of the fourth chapter. To explain, or even to illustrate, the nature of these various and complex affections, is a task of no inconsiderable difficulty.

The first eight pages are occupied with the object of shewing that some changes are not essentially inflammatory—hydrocele, for instance, or fatty tumor, or medullary tubercle, &c. So few, however, do look on these formations as the simple consequence of inflammation, that we need not play at nine-pins with the question. Mr. Travers, indeed, seems inclined to carry the view of the non-inflammatory origin of certain structural alterations, rather farther than many pathologists and surgeons. He observes, for instance, that—

"The thickening of the tunica vaginalis in chronic hydrocele, and the old irreducible hernial sac, is a process similar to the impaction and condensation of the cellular membrane in the formation of cysts; and the effusion of adhesive matter may doubtless take place under the stimulus of a slow but continued pressure in one instance, and the absorption of original structure in another, by a process quite distinct and often anticipatory of inflammation. What determines that pressure should in one case thicken and another attenuate, in one case promote the addition of a new, and in another the removal of an original texture, is the mode and degree of the local irritation applied, affecting either the nutrient or the absorbent action; this is the proximate cause; the remote cause is a final principle established in subservience to the economy, which may not improperly be termed a law." 71.

In old hydrocele, the tunica vaginalis, is as Mr. Travers observes occasionally thickened. He thinks that the change may not be inflammatory, and possibly in some cases it may not. Yet we rather conceive that, in the majority of instances, an action not dissimilar to that of inflammation will be found at the bottom of the alteration. Patients in whom this thickening exists occasionally complain of slight pain in the part, and in a thickened tunica vaginalis that we examined, there was evident arborescent injection on the surface. The capsules of certain organs, as that of the spleen, become thickened in the latter periods of life. Is this from inflammatory action? It is a difficult question to determine with precision, because it is difficult to determine what constitutes a slight degree of inflammation. If injection of the capillary vessels be considered adequate evidence of its existence, then inflammation must be granted to exist in the cases to which we are referring. But such inflammation is evidently different in kind, as in degree, from that which we commonly recognize as a powerful agent of organic change.

What determines, says Mr. Travers, that pressure should in one case thicken and in another attenuate, in one case promote the addition of a new, and in another the removal of an original texture, is the mode and degree of the local irritation applied, affecting either the nutrient or the absorbent action. A critical examination of explanations of this description, unfortunately shews, that they merely consist in substitution of a learned periphrasis for the simple expression of a fact. To say that pressure thickens a part, because the local irritation applied affects the nutrient action, is to say little more than that pressure thickens because it thickens. A real explanation would tell us *why* "the nutrient action" was affected in the one case, and the absorbent action in the other.

Our author treats in the next place of inflammation supervening on constitutional diseases. He observes, and the truth of the remark cannot be doubted, that in most cases of specific disease, as in scrofula, scirrhus, and so forth, inflammation is not an antecedent but a consequent. In fact, there are few cases of morbid alterations of structure, in which inflammation does not sooner or later come on. That inflammation is disposed to end in ulceration or in sloughing, for the vitality of morbid is not equal to that of natural structures. But though inflammation is obviously not the condition antecedent to these changes, local irritation, according to Mr. Travers, is. If such be the case, and probably it is so, we must acknowledge that in many instances all appreciable signs of such irritation are absent, and that many persons have some organ or part invaded by scirrhus, or medullary sarcoma, in whom no external symptom nor internal evidence was present to explain why it should be attacked.

"The symptoms which attend upon these chronic changes are a sensation of general malaise, languor, and lassitude, wandering pains, unequal distribution of blood and heat, uncertain rest and appetite, gradual decline of muscular tone and power, and perhaps of flesh, variable secretions, both as to quantity and kind, and an uncertain state of the temper and spirits. These are the signs of irritation from chronic change, and are indicative of the state of the system which has given origin to the morbid action, as much as of its effects. I call it, therefore, reflected irritation." 76.

Mr. Travers continues—

"The irritation of the constitution is barely perceptible in the early stage of the specific diseases. In scrofula, in all the varieties of cancer, in the numberless tumors and ulcers which have no obvious and direct local cause of origin, the constitution which has given birth to them being predisposed to their formation, discovers little sympathy with them when formed. The seeming exceptions to this observation, are those set up by violence, or whose situation interferes with the structure and function of vital organs, as tubercles in the lungs, brain, stomach, or liver, for example.

Then the irritation becomes direct, and sets up fever which rapidly proves destructive; for reflected irritation, if left to run its course, is slow in comparison with the direct, which, though originating in some purely local cause, acts, as we have seen, with such intensity as to excite the instant sympathy of the brain and stomach, and thence of the universal nervous system.

In the case of specific disease, the local change marking the accession of inflammation, is attended by an alarming aggravation of constitutional symptoms, by reason of the reflected irritation previously existing, of which the external appearance was perhaps the first clear indication." 77.

Mr. Travers is of opinion, that though a morbid poison, produced in an individual by inoculation, will sooner or later shew itself in an external form, a man may, notwithstanding, die of scrofulous or venereal irritation and atrophy, without an external or local sign demonstrating its interference with the vital economy. Such a thing is possible, yet we conceive that the proof of it would not be easy. Scrofula and syphilis are terms implying the existence of certain evident phenomena. In scrofula, for instance, there is a deposit, which possessing certain characters, is known as scrofulous. If an individual dies, and no local change is found corresponding to that which we know from experience to be scrofulous, we imagine the Sorbonne would hesitate to admit that he died of scrofula. Here we see an instance of those metaphysical possibilities in which men of talent are prone to take delight, but which the sober scheme of inductive reasoning rejects.

Mr. Travers makes a remark, in the general justice of which we agree.

" I consider," he says, "inflammation specific, in being subordinate to the specific irritation, and when this is the case, it is of a chronic character, and circumscribed as in the scrofulous white swelling, in the scirrhous tumor of the mamma, in a genuine syphilitic ulcer of the glans penis. If the action is rapid and vehement, and threatening extensive disorganization, we may be sure it is the work of some other agent in combination, as to take the last example, a state of high inflammation from neglect, or venereal abuse during the existence of sores; confinement of matter, as in perfect phymoses; intemperance, or the previous employment of mercury in frequent irregular courses during unrestrained exposure to weather. The same remark applies to venereal ulcers of the throat and external skin, to eruptions, nodes, venereal ophthalmia—take away the susceptibility to inflammation from exposure and other incidental causes during the excitement of the system by mercury, and the severe local consequences referred to the action of syphilis would be no more seen." 81.

It would be well if practitioners were generally impressed with the truth of this observation. It ought to be engraved on the mind of every professional man, that much inflammatory action during the progress of syphilis, is not immediately dependent on the syphilitic poison, but on accidental circumstances—on cold, upon excess, on the evil influence or injudicious exhibition of mercury. This is contrary to the opinions of some recent authors, but practically we are convinced of its accuracy and truth.

We have seen constitutional disease giving rise to, and mixed up with local inflammation—we are now to regard local inflammation generating constitutional disease. Yet examples of this are so frequently, so constantly presented to us, that few words are necessary to point it out. Perhaps, as Mr. Travers observes, the state of system occasionally induced by the venereal poison, is as marked and as melancholy an instance of the fact as any that could be readily selected.

The "cachexiæ" induced by scrofula, cancer, syphilis, and mercury, are reviewed in rapid order by our author. Yet we see nothing in his observations that demands particular notice upon our part, excepting, perhaps, his classification, which we give.

| "SIMPLE CACHEXIÆ. | SPECIFIC CACHEXIÆ. |
| --- | --- |
| Suppuration. | Scrofula. |
| Ulceration. | { Scirrhous Cancer. |
| Gangrene | { Lupus. Sweep's Cancer. |
| | { Medullary Cancer. |
| | { Hæmatoid. Melanoid. |
| ANIMAL POISONS. | MINERAL POISONS. |
| { Syphiloid. | { Mercurial. |
| { Varioloid. | { Arsenical. |
| &c. &c. | { &c. &c." 90. |

Mr. Travers relates eight cases of various kinds of cachexiæ. We shall select a few for the perusal of our readers.

CASE 1. *Cachectic Suppuration.* A gentleman, æt. 60, who had lived freely, had a sluggish boil at the root of the great toe. Having broken and discharged imperfectly, it was freely dilated in the direction of a hollow, which was sub-fascial and of some extent. The exposed surface suppurated, and the suppuration extended slowly upwards to the knee. For some weeks before his death, he was in a state of low fever. The peculiarity of the case was, the continual recommencement of the suppurative action ; for at the moment that the newly exposed surface was beginning to granulate, a new collection was forming higher in the limb, which became so painful as to make him anxious to have it laid open. This was done many times. The gangrenous inflammation invariably ensued upon the ulcerative, as the suppuration proceeded, so that ultimately all the parts affected were destroyed by gangrene.

Mr. Travers observes that he has seen several instances of this cachexia in the lower limbs ; but fewer in the upper. Its distinguishing character is the uncontrollable tendency to a renewal of the suppurative action in the contiguous parts, and to a decay of the healing action in that which seems prepared for it.

CASE 2. *Cachectic Ulceration.* A man, æt. 44, who had been long accustomed to drink freely and to chew tobacco, was admitted into the hospital, March 22d, 1827. " About fourteen months since, during severe weather, the lower lip became chafed at the centre, and shortly afterwards he began to smoke a pipe. The lip then swelled and inflamed, and ulcers formed in various parts of it; to these he applied different ointments, and during the summer one or two healed, while in other parts of the lip, fresh ulcers formed. The lower lip is now of a dark red color, and somewhat swollen ; towards the right side are two small jagged ulcers, near the margin, and penetrating completely through the substance of the lip into the mouth; there is some surrounding thickening, and induration. He complains of occasional shooting pains, not very severe. His general appearance is that of health.—Lot. argent. nitr. gr. v. ad aq. ℥j. ℞. Liq. arsenic. ♏. vj. Dec. cinch. ℥ij. ter diê sumend.

April 20th. The lip is much less swollen and indurated. The ulcers having gradually extended upwards and completely destroyed the fibres of the orbicularis oris, are now rapidly healing. Has not used tobacco in any form since his admission. To continue the remedies.

May 16th. Discharged quite well: scarcely any induration of the lip remaining.

In November following he returned with a fresh attack of the disease, which again yielded to similar treatment."

Mr. Travers remarks that a case closely resembling the preceding, in which the upper lip and angle of the mouth was the seat of disease, was admitted and healed under similar treatment. The subject of it was a drunkard and habitual smoker. He had a coated tongue and costive bowels, and required free purging with aloes, blue pill, and rhubarb, before commencing the mineral solution. This, after the action of the bowels is regulated, is, as Mr. T. informs us, almost a specific in such cases.

We have seen two or three instances of much chronic thickening of the lower lip near the angle, in persons accustomed to smoke excessively. The last instance that occurred to us was that of a gentleman, in whom the complaint had existed for some time. The thickening was such that the lip grasped between the finger and the thumb felt almost as thick as a large marble, the skin was red, a disposition to vesicular crust existed on it, and the part was the seat of lancinating pain. Blue pill every other night, succeeded by a saline aperient in the morning, and subsequently the alkaline infusion of sarsaparilla, effected a complete cure.

In a case of "scirrhous cachexia," scirrhous tubercles grew from the surface of the skull, from the dura mater, and in the liver; and the stomach was scirrhous throughout.

Mr. Travers relates a case of "syphiloid cachexia," which occupies thirteen pages of the work. The details are too lengthened for our purpose. An instance of mercurial cachexia is less prolix and more adapted for notice.

*Case.* Mary Pearce, æt. 34, a respectable married woman with a family, admitted June 5th, 1828, had been, for ten months, in a country dispensary. Her mouth and nose have been in a diseased state for a period of fifteen months. She has, upon repeated enquiry, invariably declared that she never had the venereal disease in any form; nevertheless she has been incessantly taking mercury; and her mouth and gums still bear testimony to the truth of this assertion.

A round excavated ulcer appears on the middle of the soft palate, and another of a similar description on the inner surface of her upper lip. Several small portions of bone have come away, whilst she was under the influence of mercury.

This patient was ordered quinine, and a lotion of the nitrate of silver. She pursued this plan for nearly a month, at the end of which time the ulceration had rather extended in the lip, and she suffered much from headache. A blister was applied to the nucha, and the linimentum aeruginis was substituted for the caustic lotion. On the 9th of July, the ulcer on the lip was extending with great pain, which prevented her from obtaining sleep at night. A pill composed of pil. hyd. gr. ij. opii gr. $\frac{1}{2}$, was ordered to be taken every other night, the quinine was discontinued, and a pint of porter daily allowed. At first, she appeared to improve a little, but towards the latter end of July, the disease was evidently assuming a more malignant character. The soap and opium pill, the application of the aqueous solution of opium to the sores, and six ounces of port wine per diem were next tried without benefit. The report states now (Sept. 19) :—The ulcer in the palate has formed a frightful excavation; a great portion of the upper lip has been destroyed by the ulcerative process; her power of articulation is in great measure destroyed; and her countenance has that pale and haggard appearance, betraying long suffering, and so characteristic of malignant disease, which this was now generally supposed to be. At this crisis it was determined to try the effect of the alterative and tonic action of mercury.

℞. Hydr. oxym. gr. iij. micæ. panis ʒj. p. conii ʒj. Aq. dist. q. s. M. et div. in pil. xxx. e quib. sum. j. ter quotidie. Extr. sarsap. ℥ss. dec. ejusdem. ℥xvj. M. cap. part. 3 m. c̄ sing. pilul. The local treatment was directed to consist in the application of dry lint to the ulcer of the lip, and gentle pressure over that with strips of adhesive plaister.

" *Sept.* 25. The effect of this trial is decidedly favorable; the ulceration has not only been arrested, but the edges are contracting: the excavated ulcer on the roof of the mouth has put on a healthier appearance, without any local application, showing evidently the beneficial effect of the medicine on the system." 114.

Her health now rapidly improved, and the ulcerations improved almost pari passu. On the 24th of October, she left the hospital well in health. There was an irregularity in the upper lip, and it did not completely meet the lower one. The pills had been diminished from three to one daily, prior to their being altogether discontinued. Prior to leaving the hospital, she again asserted that she had never suffered in any form or way from the venereal poison.

We are mistaken, if cases like these, are not regarded with interest, as examples of certain facts, rather than as illustrations of abstract notions. Setting aside all idea of direct or of reflected irritation, we will look on the last case, per se, as a specimen of what is frequently, too frequently indeed, presented in practice.

The patient had cachectic ulceration of the lip and palate, occasioning some exfoliation of bone, symptoms in short resembling those which occasionally follow syphilis, although she was not conscious of ever having contracted primary venereal symptoms. For this affection she had taken large quantities of mercury, prior to her admission into St. Thomas's Hospital. The quinine not being productive of much benefit, she was ordered mercury again, in the shape of the blue pill. Under this, the disease became aggravated, and the oxymuriate and sarsaparilla were substituted with the effect of healing the ulceration that had resisted all other modes of treatment.

Is this or is it not to be regarded as an instance of mercurial cachexia? In the first place, we should observe that Mr. Travers omits an important point in the history of the case—the mode of its commencement. How did the ulceration originate? Did the patient take mercury prior to the appearance of the ulcers of the palate and the lip, or did the latter precede, as was probably the fact, the administration of mercury? It is clear that a knowledge of such circumstances is requisite to enable us to reason on the nature of the case, yet these Mr. Travers omits to mention.

But supposing the case to have been as it was considered, and, probably, as it was justly considered, an example of mercurial cachexia, would it not be adviseable to refrain from the use of mercury in the treatment of such a case, until its employment became inevitable, that is, until all other means had failed? Cases of this sort are far from rare, and not unfrequently they are excessively intractable. In some instances, as in this, the patient is not aware of the occurrence of any primary venereal symptom. In others, though such primary symptoms existed, yet they seem scarcely adequate to account for the phenomena. A sailor, for instance, contracted simple gonorrhœa in some foreign port. He only discovered the complaint after the vessel had put out to sea. A shipmate had some calomel, and persuaded the poor fellow to take a large quantity. His mouth became extremely sore, ulceration attacked the hard palate, a great part of the palatine plate of the superior maxillary bone exfoliated, the upper lip ulcerated, and was nearly destroyed, and finally the greater part of the nose was lost by the extension of the ulcerative process.

An officer in one of the regiments employed in the Burmese war, suffered very severely from the destructive fever, that carried off a large part of our army. He took for the fever a great quantity of mercury, and his health was so broken that he was under the necessity of returning invalided to England. He had never had any venereal affection whatever. On his return to this country he came under our care with cachectic ulcers on the lower extremities, and nodes on each tibia.

In cases of this kind there appears to be little reason to doubt that the cachectic symptoms are owing to the injurious agency of mercury. The practice would therefore appear to be obvious to avoid, at all events, the medicine which has already produced such mischief. As a general rule, this is a good one—as a general principle mercury should be scrupulously abstained from in these cases. And the benefits derived from judicious general treatment are so great as to make the surgeon endeavour in all cases to give it a fair trial. Salines when any pyrexia exists—mild regimen—and good air—and when there is no pyrexia, the long-continued exhibition of sarsaparilla in various forms, combined with occasional purgatives—treatment we say, of this kind, regulated with judgment, and modified according to circumstances, will in many cases effect a cure, and in many more improve both the health and the local symptoms most materially.

But although the cachexia has been the result of the abuse of mercury, it is found in fact, whatever might be anticipated à priori to the contrary, that mercury in small doses is very often conducive to a cure, and sometimes even indispensable. Every hospital surgeon sees cases of this character. We may mention one by way of illustration.

*Case.* A policeman had gonorrhœa, which he communicated to his wife. For this he gave her, by a friend's advice, a quantity of calomel in pills. She was rendered very ill from the salivation and the depression induced by the mercury, and in the course of some weeks, vesicular eruption terminating in crust appeared upon the lower limbs. We now saw this patient and ordered sarsaparilla. The eruption nearly passed away, and the patient appeared to be almost well. Two or three months after this, the patient returned for our advice. A fresh vesicular eruption had occurred, and, the scabs separating, had left a yellow cachectic-looking ulceration, healing on one side, but spreading by a sloughy border on the other. After quieting the pain and fever which existed, by saline medicines and by opium, we again prescribed sarsaparilla. At first it had a good effect, but after a short time the ulceration spread, and assumed a very foul and unpleasant character. We now ordered small doses of the blue pill, combined with conium and with opium. The amendment was almost instantaneous, and the ulceration rapidly healed.

The most troublesome cases are those which would seem to be a compound of venereal and mercurial symptoms. The patient has had at some period a sore, for which he took an excessive quantity of mercury. Symptoms of a cachectic kind have supervened, and these are brought under the notice of the surgeon, perhaps at a later period, and in an aggravated form. It would be inconsistent with our limits and our plan to pursue this subject farther, and we, therefore, return to Mr. Travers. The fifth chapter is headed :—

*Of Constitutional Inflammations, as Examples of Reflected Irritation. Erysipelas. Cases. Gangrenous Inflammation. Carbuncle and Malignant Bubo. Cases.*

We have not yet left the subject of reflected irritation. Whether successive chapters have contributed to make its mysteries clearer to our readers, we know not ; but the present contains more observations on its nature, and further illustrations in the instances of erysipelas, gangrene, carbuncle, and bubo. We believe that most surgeons will agree with Mr. Travers, when he tells us that—

" Diseases of the constitution taking a local form are not always, as I have shewn, of an inflammatory character ; but when this local form exhibits inflammation in either of its processes, it is subjected to the influence of the constitution, and must be regarded and designated as a constitutional inflammation. The strumous ophthalmia and strumous sore throat are familiar examples. Where a diathesis prevails, an inflammation due to an obvious occasional cause, and having all the character of a simple inflammation in the outset, becomes in course of time adopted by the constitution, and modified by the prevaling diathesis ; thus catarrhal inflammation terminates in phthisis, and an excoriated lip in cancerous ulcer.

When we view such an inflammation springing up spontaneously, as an action for which we can assign no external or visible cause, we see in its simplest and most genuine character, the constitution developed in the part, which, for want of a better term, I have called ' reflected irritation,' for neither the terms sympathetic nor secondary, would have been sufficient or correct, either as descriptions or as definitions. In either of the cases above proposed the irritation is reflected, for a slight deviation from the ordinary action of the part becomes the irritant in one case, as inflammation aroused by accident is in the other. What it is, that in the absence of external appearances, disposes particular textures to particular modes of inflammation, and disposes the sex, the age, nay, the individual, to the seemingly spontaneous morbid action, is another and distinct question :—but as changes of the part often precede those of the constitution, so those of the constitution often precede those of the part, and the natural susceptibility of one to the other becomes morbid; and thus even the ordinary action of a part may be the irritant of a diseased system, as a plethoric habit aggravates some local diseases. But to justify the term 'reflected,' it is not necessary either to suppose or to deny the pre-existence of any local affection; it comprehends the local and the constitutional origin equally, requiring only a local action sooner or later, which presents the prevailing morbid type of the constitution. This may be temporary or occasional, as in many cases of erysipelas—or permanent, as in carcinoma—it may be hereditary, as in some cases of inveterate scrofula, or bred in the individual, as in others—a result of contagion, or epidemy, as in hospital gangrene, or the reverse, as in carbuncle; a poison derived, as in lues; or a poison generated in the system, as in cancer; or an affection wholly distinct from poison, either natural or morbid, as in tetanus." 119.

If we understand Mr. Travers rightly, it is necessary in some cases, in order to complete the scheme of "reflected irritation," to make the natural action of a part an irritant to the constitution. It is difficult, perhaps, to exhibit or explain this; but so many difficulties surround the whole subject of irritation, whether direct or reflected, that one more or less does not greatly signify.

Passing, then, from this speculative question to a matter of fact, we may take Mr. Travers' description of erysipelas as something definite and tangible. That practical talent, it may be homely, which consists in a close observation of phenomena, the analysis of their varieties, and the appreciation of their combinations, is of a kind that the majority of men may comprehend, and that very many are apt to admire, on account of its immediate and obvious results. It is this sort of talent which has done so much in the discrimination of disease—which has exploded the old notions of white swelling and asthma, and a host of other unmeaning generalities. It dives no deeper than the physical qualities of things, and leaves the abstraction of processes, and sympathies, and so forth, to other inquirers and to other intellects. The thinkers of this school may be termed the utilitarians of science, and they at least enjoy no mean advantage—that of being understood. We confess, on our own parts, a leaning to this description of utilitarianism, and we, therefore, gladly, turn to the practical subject of erysipelas.

Mr. Travers observes that the parts disposed to erysipelas are, first, the skin—secondly, the skin and subcutaneous cellular membrane—thirdly, the mucous and serous membranes.

1. *Erysipelas of the Skin.* This is the slightest form of the disease; slightest, that is, in point of structural implication, for sometimes it proves fatal, and frequently it is severe. The description of it is so familiar, that we need not insert it here. As Mr. Travers states, the erratic form of the disease, or its appearance in different regions of the body, or in distinct patches, with sound interspaces, at the same time or in quick succession, is more serious than where the erysipelatous redness is continuous. It shews a debilitated state of constitution, and, generally speaking, tonics are more necessary for this than the other description.

2. *Erysipelas of the Skin and Cellular Membrane.*

"When the superficial appearance described above is attended by swelling from a serous effusion into the cellular membrane, it is distinguished by the term 'œdematous.' If, as happens from a variety of causes, this serous effusion is unaccompanied, nay, unpreceded by the inflammation of the surface, it is not erysipelas but œdema. The œdematous is the most common form of the disease, and is always important, but seldom dangerous, except when situated about the head, when it is always more or less so. The œdema is a mode of relief to the overcharged vessels, which either averts or stands in the place of suppuration, or facilitates it, according to the state of constitutional power. Thus it is a very frequent termination of the disease." 123.

We do not exactly perceive the gist of Mr. Travers' distinction between œdema end erysipelas. Œdema, according to him, merely wants redness of the skin to be erysipelas. But surely they are altogether separate things, œdema being merely effusion of the serous part of the blood, from pressure on the veins or from debility, while erysipelas is essentially an inflammatory affection. The two affections are so totally dissimilar, that we see no advantage in the institution of a comparison of any sort. There is no possibility of their being confounded. But to proceed.

"When from the violence of the action, or the loss of tone of the absorbent system, the effusion becomes excessive, the disease terminates by a process of suppuration, and one large diffused collection, or several smaller distinct collections of pus are formed in the cellular texture, which is spoiled and sloughy. The matter is of a thin consistence, secreted by the same vessels which have loaded the cells with serum under a continuance of the inflammatory action, an action incapable of the higher stage of adhesive deposition. The cellular texture dies, and with the effused fluids undergoes that state of decomposition to which the expressive term 'pourriture' is applied by the French. The entanglement of the matter by the shreds of dead cellular substance, renders it impossible to discharge such collections but by free incision, and the best practice is to incise them as soon as the suppurative process is manifested, by which their further extension is prevented and much relief obtained. As such collections are not indicated by what is called 'pointing,' the presence of pus is not always ascertained so early as is desirable, and it is sometimes not until matter is formed in great quantity, so as to fluctuate, that the surgeon is aware of its existence. This is a sort of delusion conveyed by the œdema under cover of which it forms.

The third termination of erysipelas is in gangrene—in which the skin dies along with the cellular membrane. This is rare, and happens abruptly and from purely constitutional causes; unless a result of excessive distention, as in enormous fascial abscess, or of disorganization from violence, as in bad compound fracture. It is remarkable however to what an extent the destruction of the subjacent membrane, following the inflammation and excessive distension of the skin, will proceed, without affecting the vitality of the latter. Upon the eyelid and prepuce, where its texture is peculiarly delicate, we oftenest see it die.

Gangrenous erysipelas is distinguished from gangrenous inflammation by the prior existence of the erysipelas, and its confinement to the integument, though diffused over a large extent of surface, as the entire or the half of a limb, for example. In gangrenous inflammation there is no such diffused swelling and redness as belongs to erysipelas, prior to the appearance of the phlyctenæ or sphacelated spots. When actual death has taken place in erysipelas of the trunk or limbs, the signs of distinction become faint and would vanish altogether, but that the extent and circumstances of origin of gangrenous erysipelas seldom permit of the maintenance of life long enough to observe the phenomena peculiar to it.

In the terminations of erysipelas above mentioned the adhesive process is passed over altogether, and this absence of the boundary which, like the hoard erected round a building under repair, precludes interruption to and from its neighbourhood, constitutes the strongest local character of erysipelas. Suppuration, when not so circumvallated, is an action without an object and as purely destructive as gangrene. For not only is the action without local check or confine, but it is also destitute of the material of repair, organisable lymph, the basis of granulation. I say organizable lymph, that which is essential to phlegmon and abscess, because the term phlegmonoid has been adopted to designate a variety of erysipelas, in which the effusion is of firmer consistence than the œdematous and suppurative, yielding less to pressure, and conveying no sensation of fluidity to the fingers of the examiner. The distinction is founded in truth, but the term must not be understood to convey that the matter effused is an organisable medium. In this sense we might as justly call carbuncle, phlegmonoid. A concrete albuminous matter, such as is seen after puncturing and emptying the vesicle of a powerful blister, is seen upon incision of this phlegmonoid erysipelas when the serum has drained away from the cells, which are distended by it to their utmost capacity—and with this, puriform matter, and in an advanced stage, sloughs are here and there intermixed." 126.

The two preceding quotations present the whole of Mr. Travers' description of erysipelas, so far as the affection of the skin and cellular tissue is concerned. The separation between the simple cutaneous erysipelas, and diffuse inflammation of the cellular tissue is wide enough and clear enough to prevent much confusion in practice. But the two are not always so distinct, for they run into each other, and numerous intermediate forms exist, some approximating more closely to the cutaneous erysipelas, and some to the cellular inflammation.

The broad line of demarcation between the two affections is this:—that, in erysipelas, the inflammation begins in the skin, and affects the cellular tissue, consecutively—while in inflammation of the cellular tissue, *that* is first involved, and afterwards the skin.

In the simplest form of cutaneous erysipelas, that form which bears a very close resemblance to the exanthemata, the cellular tissue is scarcely, or even not at all involved. The disease runs its course in a week or ten days, and terminates in desquamation of the cuticle. Yet even in this, some serous infiltration usually takes place in the subcutaneous cellular membrane, especially in that which contains no adeps, and after the subsidence of the erysipelas, small abscesses are often found in the situations we have mentioned.

In the next nosological grade of erysipelas, for though we leap from one distinct resting place to another, the advance of nature is more uniform and less abrupt, we find the cellular tissue more involved. The erysipelatous inflammation of the skin, characterized by the definite margin, is combined with much effusion into the subcutaneous cellular texture. This effusion is usually a combination of serum and lymph. It is serum and lymph in the commencement, but ultimately the lymph is almost always replaced by pus or mingled with it, and in a limb or a part of the trunk thus affected, we find in the latter stage of the affection the cellular tissue presenting here and there collections of matter, varying in size and in diffusion according to the severity of the attack and the constitutional vigour of the patient. If Mr. Travers intends to imply that the suppuration in the cellular tissue, even in this severe form of erysipelas, is never strictly limited, we cannot altogether agree with him. We have seen collections of matter as circumscribed in erysipelas as in any other form of inflammation. The fact is, that much depends upon the patient's strength, and very much indeed upon the treatment. Judicious support, and the happy exhibition of stimulants and tonics, will often arrest the diffusive character of suppuration, and enable the constitution to limit it by a boundary of coagulated lymph. We may make one observation which we think will be found to be generally correct. Wherever, in the early stage, or during the progress of erysipelas, the hardness in the subcutaneous cellular membrane, arising from effusion of lymph into its texture, is perceived, there the surgeon may count on afterwards finding an abscess. In fact, whenever there is much effusion, even though the quantity of lymph be small, more or less suppuration almost always follows, and in elderly persons, or in those of enfeebled constitutions, not only is the suppuration extensive, but the skin also frequently dies.

Diffuse inflammation of the cellular tissue is allied to the form of erysipelas last mentioned. There can be little doubt that what has been termed erysipelas phlegmonodes, is really diffuse inflammation of the cellular tissue. This affection is a common consequence of injuries of the lower extremities, especially in the large metropolitan hospitals, and it forms the most serious and most falal result of lacerated wounds and of compound fractures. We have watched these cases closely, and the following appears to us a pretty accurate account of the pathological phenomena and conditions which they present.

In a limb affected with diffuse inflammation of the cellular tissue, in a severe degree, there is first observed tumefaction of the limb in the vicinity of the wound. This tumefaction is evidently seated in the subcutaneous tissue, for it pits on pressure, although the pitting is not so deep as in mere œdema. Sometimes the part feels doughy rather than pits on pressure, sometimes it feels quite firm and resisting, sometimes it crepitates, in consequence of air being effused with the serum and lymph in the tissue. The skin of the swollen part displays some discoloration. Sometimes it is faint and rather florid, sometimes it is dark and more approaching to purple, more frequently in bad cases it is brownish and mottled. The margin generally *is*, but not unfrequently *is not* defined, and the redness of the skin never passes beyond the swelling in the subcutaneous cellular tissue, and commonly does not extend so far. With the progress of the affection, the swelling spreads up the limb, and the discoloration of the skin accompanies, or, rather, follows it. As the swelling increases the skin becomes stretched, it grows darker in hue, perhaps vesications are observed upon it, it sloughs, and the subcutaneous cellular tissue is discovered in a mixed state of suppuration and of sloughing, to an extent that a person unaccustomed to the affection would probably not suspect. Such are the phenomena of the disease displayed on the surface of the limb.

If we trace its characters by dissection, we find them to be these. In the earliest stage there is a quantity of bloody serum effused into the subcutaneous cellular texture, which is evidently more vascular than natural. Over this the skin is little if at all discoloured. This proves, of course, that the disease commences and has its essential seat in the cellular tissue. At a more advanced period, the vascularity of the cellular membrane is lost, and the bloody serum is replaced by a deposition partly of pus and partly of lymph, which fills the cells. Over this the skin is decidedly affected, discoloured deeply, perhaps vesicated. Finally, the cellular tissue, distended by the deposition we have mentioned, is in a state of slough, and looks, as an author has well described it, like tow soaked in purulent matter. The skin may still retain its vitality, for some little time it usually does so, but sooner or later it dies, its death however being always consecutive to that of the cellular membrane below it.

This is a brief, but, so far as it goes, a correct account of the phenomena and pathological characters of diffuse inflammation of the cellular tissue, or phlegmonoid or gangrenous erysipelas, as it has been called. That it is not essentially seated in the skin must be owned by all who attentively study its phenomena, and that the principal interest, practically speaking, is connected with a knowledge of the state of the subcutaneous cellular texture, must, we think, be acknowledged also. In simple erysipelas, incisions are not necessary; in this affection they are absolutely indispensable. They are indispensable for the preservation of the skin, which, stretched by the effusion in the cellular tissue beneath it, and deprived of its vascular supply by the strangulating deposition in its cells, must inevitably die, unless the distension of the cellular tissue be removed, and its deposition allowed to escape by free incisions into it.

We must admit that it is difficult to say in all cases that this affection has not an erysipelatous character. But we must say also, that in the greater number its seat is unequivocally the cellular membrane, in which it begins, in which it runs its course, and to the state of which our attention must always be mainly directed.

We may now return to Mr. Travers. He adverts to erysipelas of the reflected mucous membrane.

"The conjunctiva, membrane of the fauces, of the air tube, of the rectum, and I believe, of the pulmonary and intestinal surfaces throughout, are occasionally the seat of this species of inflammation. It is rare by comparison with that of the external tegument, and perhaps chiefly occurring in the immediate vicinity of the skin, as where that membrane is reflected or modified by its relation to the function. The subjacent cellular tissue is involved in the inflammation, giving it the œdematous character, and it is probable that this tissue, pervading and connecting all the textures of the body, is in all liable to erysipelas, though not hitherto presenting itself to observation in such a form as to be generally so recognized.

The excessive chemosis of the muco-serous membrane of the eye, which ushers in the acute suppurative ophthalmia, indicates, not less than its disposition to gangrene upon the cornea, that it belongs to this species of inflammation. In the pharynx and membrane of the glottis it arises from cold and causes operating constitutionally, in which it sometimes turns gangrenous, as in scarlatina maligna; and from wounds of the throat, or caustic substances applied or swallowed, the obstruction to respiration from excessive swelling is sometimes such as to demand tracheotomy to prevent suffocation. It is also sympathetic, more or less, with acute œdematous erysipelas of the head and face, in which disease the patient is generally from this cause deprived of the use of his natural senses of smell, taste, and hearing. The contagious property belonging to erysipelas is strikingly manifested in some of these cases. I have known it fatal to three of the same family within the space of ten days. In the rectum ligatures of internal piles, and of bleeding vessels, necessarily including a portion of the sub-mucous membrane, give origin to this inflammation; and I have seen it in several cases produced by the division of a fistula, in which erysipelas of the nates, and gangrene of the integuments surrounding the anus terminating the erysipelas, terminated also the patient's life. The peritoneum is exposed to this species of inflammation after child birth, and I believe that a large proportion of the puerperal cases are cases of erysipelas. The diffusion of the inflammation over the cavity, the non-production of adhesions, and the presence of flakes of albuminous matter floating in a curdlike serum, the sudden prostration of power, and the strongly marked property of communication, whether by contact or atmosphere, lead me to consider it as of this description. The absence of œdema is explained by the strictness of the sub-serous cellular texture. It is probable that the connecting cellular tissue in the interior of all structures, whether muscular, membranous, nervous, vascular, or bony, is subject to be so affected in those painful diseases which, under the denomination of chronic rheumatism and neuralgia, so frequently defy all our ingenuity of explanation and of treatment.

And yet more certainly we may conclude this to be the case in those deep-seated abscesses, necroses, and tumors hard and soft, which are accompanied with great bulk and consequent extension of all yielding textures, so as to threaten life as well as limb." 128.

It appears to us that Mr. Travers makes use of the term erysipelas in a very loose sense. At one time he employs it to designate a particular inflammation of the skin, resembling the exanthemata, and exhibiting certain well-known phenomena—at another he seems to make it represent a spreading inflammation of any description. This may be convenient, but is very lax. Mr. Travers, for instance, affirms that the puerperal inflammation of the peritoneum is frequently only erysipelas. To compare the former to the cutaneous exanthema would be obviously absurd; the comparison therefore must refer to erysipelas as a symbol merely of a spreading inflammation. Yet this is a substitution of words for words—it means nothing, explains nothing, and leaves matters just where they were. In this, as in many other instances of medical reasoning, we are forced to regret the disregard of the logical rule—to define strictly the terms we use, and to use the terms as strictly as they are defined.

We have seen some interesting cases of erysipelas spreading down the throat, and extending up the vagina and the rectum. We will briefly mention the particulars of one or two.

*Case* 1. A patient in St. George's Hospital had erysipelas of the head and face. About the sixth day the erysipelas became suddenly much paler, and the patient complained much of sore throat. On examining the throat, a dull red blush suffused the fauces and palate. The patient became delirious and was very low. In a day or two after the commencement of the affection of the throat, dyspnœa with cough, and obscure pain in the chest, supervened. There was the mucous rattle of bronchitis in both sides of the chest, and imperfect respiration in the lungs. In two or three days the patient died. On examination of the body, a slight vascularity was found to extend down the pharynx and into the œsophagus, and to spread down the larynx and trachea to the bronchi. The mucous membrane of the latter was slightly injected, and much mucus was contained in the tubes. The lungs were congested, and partially œdematous. We suppose that this must be viewed as an example of erysipelas extending down the air-tubes to the lungs.

*Case* 2. A girl in the same hospital had strumous corneitis, and some other affection which we do not at this moment recollect. She was attacked with erysipelas of the face, at a time when it was very prevalent in the wards. After a few days she was seized with sore-throat, and the palate was seen irregularly inflamed. This was followed by obstinate vomiting, and dull pain in the epigastrium on pressure. Purging followed, and the patient appeared to be sinking under severe gastro-enteritis. About the ninth day after the commencement of the erysipelas upon the face, an erysipelatous blush was observed to surround the anus; from this it spread over the nates, and desquamated. It was spreading on the nates at the time of its desquamation on the face. When the erysipelas had become established on the nates, the symptoms of gastro-enteritis subsided. The patient recovered. This case excited much interest at the time. It appeared to be an instance of erysipelas, extending through the whole length of the intestinal tube. Be that as it may, the facts were as we state them.

*Case* 3. A girl was in the hospital, with a sore upon one nympha. After the application of caustic, erysipelas appeared upon the corresponding labium. It spread to the groin and beyond the anus, for a few inches upon the nates, where it stopped. But it also spread up the vagina, which, so far as could be seen, was dry, and presented a distinct erysipelatous redness. The patient rapidly became very low, and complained of dull pain in the hypogastrium, with tenderness on pressure in the pubic region. She had also great pain in micturition. Vomiting succeeded, and, in five or six days from the first appearance of erysipelas upon the labium, she died. On examination of the body, the mucous membrane of the vagina and of the uterus was found irregularly vascular; pus was contained in the cavity of the latter. The mucous membrane of the bladder was partly vascular and partly ecchymosed in patches. The peritoneum of the pelvis was inflamed, and purulent matter was found here and there in its recesses. The erysipelas, in this case, obviously extended from the orifice of the vagina, along that canal to the uterus and bladder. The inflammation of the peritoneum, on their exterior, must have resulted from the extension of inflammation, by continuity of tissue.

We might mention other cases, but the foregoing are sufficient. They are interesting as examples, fortunately not common ones, of the extension of erysipelas to the various mucous membranes, and of the consequences to which such extension may give rise. Occasionally, as Mr. Travers observes, erysipelas spreads up the rectum, in consequence of operations performed on the lower extremity of that gut, and this is not unfrequently a fatal accident.

We are not disposed to agree with Mr. Travers, in his supposition of the probability of the cellular tissue, pervading and connecting all the textures of the body, being in all liable to erysipelas, though not hitherto presenting itself to observation in such a form as to be generally recognized. That the inflammation of erysipelas may extend to any portion of the cellular tissue, we entertain no doubt; but we greatly doubt the advantage of stretching the application of the term erysipelas so far, as to make it include all diffusive inflammations. We may call such erysipelatous if we please; but we think that the term diffuse inflammation is wide enough to include the facts, and liable to no misconception nor obscurity. The study of the phenomena of diffuse inflammation of the cellular tissue, in the different regions of the body, is interesting and important in a high degree. It has not yet received the attention which it merits, and it requires to be viewed as a substantive affection, not as a variety of erysipelas.

Mr. Travers draws a line of distinction between erysipelas and other inflammations of skin, which not unfrequently receive that name.

"Erysipelas is constantly confounded with inflammations of the integuments, which arise under different circumstances, and are of a distinct character. Thus the inflammatory œdema so commonly following the bites of leeches on the eyelids, prepuce, scrotum, and other cellular textures, parts much disposed to erysipelas; the sympathetic erythema, or even suppurative œdema, which takes possession of the integument of a limb, in inflammation of the fascia, veins, or absorbents, and in sub-fascial collections of matter; and the sympathetic blush of the skin covering a gangrene, as of the tunica vaginalis testis, or a mortified hernia, or a carbuncle, are denominated erysipelatous, by which it is intended to designate an adventitious form of the disease. The state of the integument which results from infiltration of the cellular membrane by an extravasated fluid, from sympathy with inflamed absorbents or veins, and from changes of the subjacent membranes, or accumulations beneath them, is widely differing from erysipelas; and we should greatly reduce the list of cases classed and reported under this head in surgical practice, if we would confine the term to those in which the skin, or skin and cellular membrane, are primarily affected.

If a portion of integument like the prepuce or eyelid swells, reddens, and, instead of simply suppurating and ulcerating, a central portion turns gangrenous and separates, leaving an ulcer penetrating the integument—a very frequent case—it is a misnomer to call it erysipelas. It is inflamed œdema, a disease altogether local.

Serous and purulent effusions in the cellular membrane are frequently met with, independent of any discoloration of the skin, as in many of the cases lately described under the term of 'diffuse inflammation of the cellular membrane'—and in the rapid œdema from substances acting as poisons on the stomach as well as in anasarcous swellings. Commencement in the tegument, external or internal deficiency of adhesive inflammation, and a peculiar state of the system with which the inflammation is connected, whether spontaneous or the result of injury, are diagnostic characters of erysipelas." 130.

The sympathetic blush of a carbuncle, and inflamed œdema, are odd terms. In carbuncle, the subcutaneous cellular tissue is inflamed and dies, and the skin covering the inflamed cellular tissue is, as in all instances of inflammation of that tissue, inflamed also in a less degree. This, of course, is not erysipelas, yet it has almost as good a right to receive that name, as in the case of diffuse inflammation of the cellular tissue, when it commonly receives that designation. What inflamed œdema is, or, indeed, what the term can mean, we confess we feel uncertain. Œdema is a serous infiltration of the cellular tissue, and it is difficult to support the philosophical exactness of calling an infiltration inflamed. An inflamed tissue is sufficiently intelligible—an inflamed fluid effusion is much less so.

Mr. Travers having disposed of the *physique*, proceeds to the *metaphysique* of erysipelas. He likens it to tetanus, and our readers will see how.

"In what does the local disease consist? Inflammation, indicated by a preternatural fulness, color, sensibility of a portion of the skin derived from the injection of its vessels with red blood, vessels which are in health transparent, and carrying only the colorless part of the blood; which, although capable of being affected by the permanently increased action of the heart, are nevertheless so far remote from, or independent of its ordinary influence, as to be unaffected by the state of the heart's action, and to carry on actions and processes without any obvious sympathy of the heart, as evinced by the general circulation. The sympathies of the skin are peculiar in being more direct with the brain than with the heart—the blush precedes the sensible increase of the heart's action, if any be perceptible, as the pallor precedes the state of syncope. So, also, the fever of scarlatina, measles, and small-pox precedes the eruption, and the inflammation of erysipelas precedes the fever. Such an interval not peculiar to erysipelas, but applying also to cutaneous inflammation from burns and scalds, as well as the injuries of cutting, tearing, and bruising the surface, tends to shew that the sympathy is slowly evinced between the heart and skin, as compared with that existing between the latter and the nervous centre. Hence I consider erysipelas to be a nervous inflammation, bearing to the actions of the vascular system, that irregular and undefined relation, which tetanus has to the actions of the nervous system. And it is remarkable, by the way, how the injuries are in their nature assimilated, which tend to the production of one or the other of these diseases." 131.

This pathology is somewhat transcendental, yet it may not be any thing the worse for that. But the whole, in this instance, is built upon a fact which we really feel some hesitation in admitting. Mr. Travers affirms that the fever of scarlatina, measles, and small-pox precedes the eruption, but the inflammation of erysipelas precedes the fever. Now this is just the point on which we believed that pathologists took their ground, in assimilating erysipelas to the exanthemata—we always imagined that the fever of erysipelas preceded the inflammation. We have seen a good deal of the disease, and certainly we have usually seen the fever usher in the eruption. Such a case as this is not uncommon. A man receives a scalp wound—on the third or fourth day he has a rigor, succeeded by decided fever. The surgeon is apprehensive of inflammation of the membranes of the brain. In 24 hours, sometimes after a much longer interval, erysipelas of the scalp appears. We have seen, on more than one occasion, the surgeon bleed the patient profusely, under the idea that inflammation of the brain was present, when the supervention of the erysipelatous eruption proved, that the fever was only its precursor.

It is much more easy to designate erysipelas "a nervous inflammation," than to explain what a nervous inflammation is. We, therefore, refer the curious reader to the pages of the work itself for further observations on this subject. We may, therefore, allude to a more intelligible point—the causes of the disease when idiopathic.

"Of idiopathic erysipelas the causes are referred to sudden changes of temperature, a humid soil and atmosphere—of which, particular conditions at uncertain periods of the year, render the disease more frequent. Especially also, an unchanged, and therefore, as well as from other causes, a polluted atmosphere; such as the ill-ventilated apartments of the poor, and even of many old parochial and eleemosynary institutions, manufactories in crowded cities, narrow allies, and underground chambers, where running sores, and other uncleanlinesses additionally contaminate the stagnant air.

Indigestible food, exciting or depressing passions of the mind, excessive fatigue, or habitual indolence, coupled with a torpid and vitiated state of the great secreting organs ; luxurious living, and the habitual use of fermented liquors to excess, are among the causes which predispose to it." 133.

Thus we see in this, as in other instances, that causes the most opposite give rise, apparently, to the same effect. Heat and cold—fatigue and indolence—high living and low living, are ranked by our author as the causes of the disease. A category so wide would rather prove our ignorance than knowledge. Perhaps the general expression that approaches most nearly to the truth is this—that whatever deranges or checks the secretions tends, in certain habits, to give rise to erysipelas. This is vague enough, but its very vagueness is requisite to enable it to comprise all the facts.

When we descend to more particular, and less universal causes of the malady, damp and cold air may probably be ranked among the principal, if not the first. It is well known, at some of our London hospitals, that erysipelas prevails during a continuance of easterly winds, more than under any other atmospheric condition. It has always been remarked, at one of those institutions, that erysipelas was common when sore-throats and catarrh were common also, and that both made their appearance when sudden changes of temperature took place, or when, as we have observed, the wind continued for any length of time to be easterly.

After some remarks on the reasons why it should attack the persons that it does attack, and why, being a disease of debility, it should invade, very shortly after accidents, persons who appear to be any thing but weak, Mr. Travers sums up his opinions in this manner.

" My own opinion is, from careful reflection on its history and phenomena, that erysipelas derives its local peculiarities from those of its seat, viz. the membranous capillary circulation ; and from the intermediate influence of the nervous system between this and the heart, and all the other organs of the body, its peculiar pathology." 138.

In other words, erysipelas derives its *local* peculiarities from its *local* situation, and its pathology from the intermediate influence of the nervous system, between the capillary circulation and all the organs of the body. If reflection has led our author to nothing more clear nor more definite than this, we fear it is an instance of the chiaro-scuro, more adapted to excite admiration than to edify.

It has generally been conceived, that the skin sympathises more closely and immediately with the pulmonary and the gastric mucous membrane than with any other tissue or with any organ. But Mr. Travers believes that the sympathy of the skin and brain is more intimate than those that we have mentioned.

" Its sympathies (the sympathies of the skin) are universal, but the brain is the organ of sympathy, and it were as idle to look for any other medium of communication between the skin and the heart, the lungs, the stomach or the kidney, as to renew the search for a duct extraneous to the circulation between the two last-named organs, to explain the rapid transition of fluids. The skin is the organ, consequently, upon which a large proportion of the diseases to which the body is liable, make their attack—not so much by actual ingress as by disordering or arresting its function. Such is the commonest origin of fever, of visceral inflammation, of glandular, and many other diseases. It is remarkable in how many of its own diseases it suffers secondarily, or by sympathy with its fellow organ, the interior skin, or lining membrane of the visceral cavities and alimentary canal. With the pulmonary surface the skin sympathizes in the noxious effects of atmosphere and temperature in all their qualities and vicissitudes, with the heart in its varieties of circulation from a thousand causes, with the stomach in the quality and quantity of its ingesta, and remarkably with the kidney, so as indeed, in some instances, almost to reciprocate its functions. But with none is its sympathy so active and so instant as with the brain, of which, in a certain sense, it may be regarded as a production." 139.

It is perhaps not quite certain that all sympathies between organs travel through the brain. The spinal marrow and the ganglionic system may probably do a little work in this way, and the experiments of Le Gallois, under the search for any other medium of communication than the cerebrum, may not be absolutely idle.

It would undoubtedly be absurd to deny the influence exercised by the nervous on the vascular system. Not to mention other instances, a familiar one exhibits the fact in a homely yet still in a striking manner. The blush, occasioned by the vascular suffusion of the cheek, is instantaneously produced by the agency of a thought.

Mr. Travers observes that all the predisposing causes of erysipelas are, in their nature, debilitating, while its exciting causes are such as irritate enough to produce inflammation. Does it ever, says he, appear either as a spontaneous affection, or as a result of injury in a sound condition of the system? He answers the question—never.

"The symptoms, as well as the causes, are such as characterise deficient power. In addition to those of passive congestion in the vessels of the brain,—as pain and a sense of weight extending throughout the body and limbs, confusion, lethargy, deafness, or wandering delirium,—frequent retching, which it is often difficult to appease, and a degree of prostration resembling that of typhus, which incapacitates the patient from moving in his bed, are symptoms of erysipelas, though confined to the extremities. A surcharged state of the veins and sinuses of the brain, and a quantity of fluid beneath the arachnoid tunic are the principal, if not the only, recent changes observed in post-mortem examinations.

The apoplectic character is still more strongly marked when the disease affects the head. The patient is scarcely to be roused from his stupor, and the breathing is stertorous. I have seen maniacal delirium present, but it is rare compared with that of dreaming and muttering. All accidents accompanied with shock or concussion of the brain, or inducing an undue determination of blood to that organ, and especially wounds of the face and hairy scalp, are particularly prone to erysipelas. Indeed, it is a consequence of scalp wounds almost invariably, when closely bound up by strips of resin plaster, to promote union. The practice is highly objectionable. On the other hand their edges should not, if clean incised wounds, be permitted to gape, as this promotes a diffused suppuration of the cellular membrane. The agglutinative should be unirritating, and the daily dressings light, with interspaces for the discharge. Sutures should never be employed." 144.

We do not agree with Mr. Travers in his unconditional condemnation of sutures. There are many kinds of scalp-wounds, in which it is impossible to keep the flaps of integument approximated without the application of a suture, and less pressure is necessary in the subsequent dressings if a suture is judiciously employed. As a matter of fact, we have frequently used one with advantage, and although it would be wrong to recommend their indiscriminate use, yet it is questionable if they ought to be totally proscribed.

The treatment of the disease is considered by our author at some little length. He believes that the best plan lies in medio between the two extremes of active depletion and active stimulation. As a general rule, there can be little question that this approaches the truth more nearly than any other. But, like all general rules, it admits of exceptions, and the rational surgeon would not select some treatment intermediate between the furious blood-letting advocated by Mr. Lawrence and the bark of Dr. Fordyce, and apply this to all cases of erysipelas that came before him. The robust countryman and the squalid London gin-drinker neither exhibit the same symptoms, nor can reasonably be deemed subjects for the same kind of treatment. In the first the pulse is full, the tongue white, the tint of the erysipelas florid—in the second, the pulse is weak, the symptoms verging towards prostration or typhus, the tint of the erysipelas pale or bluish. Such phenomena can never indicate the same remedial means, and it is as rational to endeavour to explain away their difference by a miserable joke upon milestones, or by a query where town ends and the country begins, as it would be to deny that a difference exists in the water at London Bridge and at the Nore, because no waterman could exactly point out the line of separation between the salt-water and fresh.

There are two great practical precepts in the management of erysipelas, which can seldom, if ever, prove fallacious—the first is, to endeavour to re-establish healthy secretions—the second, to be guided in the use of tonics or depletion by the general condition of the patient. In the cutaneous erysipelas, and in that in which the cellular tissue is involved, the first five or six days, or even more, are occupied with the increase of the eruption. During that period there is generally a good deal of febrile disturbance, and the symptoms are more or less inflammatory. From the sixth to the eighth day the eruption has usually attained its height, and part or the whole of it begins to desquamate. The constitutional symptoms at that period exhibit a remarkable change. The patient suddenly becomes much lower, nay, he may abruptly fall into a typhoid state. It is for this period and for this stage that the surgeon should anxiously watch. Prior to it, aperients to improve the alvine secretions and salines are in most cases the best remedies. They assist and they do not interfere with the operations of Nature. But so soon as the patient begins to exhibit the symptoms of depression, the surgeon must turn round and promptly administer support, if not stimulants. It should be recollected that this change occurs rather suddenly, and the patient, who the day before may have seemed in a condition of vigour and of active pyrexia, is found, perhaps, with scarcely any pulse at the wrist, and in a state bordering on, if not actual prostration.

Mr. Travers next touches on the practice of incisions. This he lauds, yet not more than it deserves. The proper employment of incisions is certainly one of the greatest of modern surgical improvements. It has saved both limb and life. The incisions should be sufficient to effect three objects—to relieve the tension of the skin—to evacuate purulent matter—and to expose and give adequate vent to the sloughs of cellular membrane. These are the objects to be held in view—the exact dimensions of incisions can never form the subject of any certain rule. Yet those of inordinate extent must certainly exhaust the powers of the patient without affecting any corresponding good.

"The incisions may be proportioned to the mischief to the extent of three inches, but seldom need or should exceed that space. They should penetrate the entire cellular structure, and if arteries of a size requiring more than the pressure of the finger for two minutes be divided, they should be secured by ligature. The venous bleeding is beneficial; arterial is neither advantageous nor safe, as has been sufficiently proved.* Two, three, or more such incisions may be made, if an entire limb is affected. Of the puncturing practice I have no favorable opinion. It irritates, without effectually unbinding or unloading, and is a cruel prolongation of suffering, especially when the disease is situated on the face and head." 148.

We cordially agree with Mr. Travers in these sentiments. From what we have seen of the "puncturing practice," we are inclined to think that, in severe cases, it is inefficient, and, in trivial ones, unnecessary. We are bound to believe, from the good opinion entertained of punctures by surgeons, whose judgment is undoubted, that they are occasionally beneficial, but, so far as we have seen, we cannot say that much advantage has arisen from them.

The last point connected with this interesting, because common and severe complaint, is its contagiousness.

"The proofs of the contagious character of erysipelas are more decisive, and unfortunately more abundant, than of almost any disease with which I am acquainted. When it prevails in a particular ward of a hospital, cases of accidents, and even trifling operations, should, if possible, be excluded from it. A patient, the subject of wound of any sort, should not lie contiguous to one affected with erysipelas. The idiopathic arises from the traumatic, as this from the former, and each from the same, as I have repeatedly seen. The practice of scouring the floors of wards and ships' decks has been supposed to contribute to its production, and the substitution of dry-rubbing the floors and decks, to relieve the ward and ship of the contagion. This practice, Sir Benjamin Brodie informs me, has been successfully employed at St. George's Hospital in wards so affected.

It is not to my purpose here to enter into this subject in detail, or attempt to analyze the character of the contagion, but I may observe, that it is an additional corroboration of the argument, that the disease holds that peculiar association which I have described with the nervous system; this being the organ by which the deleterious agency of the matter of contagion is first manifested, whether admitted by the cutaneous or the pulmonary surface. And the history of all the local contagious diseases agrees in shewing the nervous system to be primarily engaged in them to a degree over-proportioned to the vascular, whether plague or small-pox, scarlatina, or puerperal inflammation, or, lastly, as being the medium by which they prove destructive." 150.

Thus we see that Mr. Travers is a contagionist, *ad extremum.* If the proofs of the contagion of erysipelas are more decisive and more abundant than those of almost any disease, for instance, measles or small-pox, our author must have been more fortunate than the majority, even of contagionists. Erysipelas prevailed to a great degree in St. George's Hospital a few years ago. The question of contagion was examined very

---

* "If such patients are left but for a few minutes to bleed, they are left to die, and the object and end of this invaluable practice are misunderstood. A needless extent of incision not only increases this danger, but is open to other obvious objections."

carefully and closely, yet the proofs were far from numerous or satisfactory. We have seen a good deal of the disease ourselves, yet we feel great doubt in admitting that we ever witnessed one unexceptionable instance of contagion. We are not particularly sceptical in this respect, yet such is our sincere impression.

Our author relates eight cases of erysipelas. They present no features of peculiar interest, with the exception of one, which is instanced as an example of the disease affecting the mucous membrane of the throat. This we shall insert.

*Case.* " In the year 1819, a poor woman called to consult me under great distress of mind, having lost her husband a very few days before from a sore-throat. Except a general redness of the fauces and slight tumefaction, there was nothing to create alarm, but her husband having died of the same disease after a very acute illness,* over which the remedies prescribed had no control, I entered into her apprehensions, and directed a copious venesection, blistered the throat, and ordered calomel and antimony at short intervals. At present she had no dyspnœa, but a difficulty in swallowing. I desired her to get away from her home, which she did for two days, but having a family at home, and finding the difficulty of swallowing increase, she returned, took to her bed and in two or three days died, under symptoms exactly similar to those of her husband. There was high fever and excitement to convulsion in the attempt to swallow, great tumefaction and irritability of the fauces, and lastly suffocation from impeded respiration, which took place only a short time before death. In consequence of my being absent, Mr. —— was called to her assistance and performed the operation of bronchotomy, but without relief. On examination after death, I found the cricoid cartilage had been only divided, and that the edges had fallen together again, so that no permanent aperture existed. The inflammation was confined to the fauces, pharynx, and rima glottidis, the membrane being so tumefied by infiltration, as to prevent the passage of food, and lastly of air. A child was threatened with the disease, but by immediate removal to the hospital the inflammation was cut short before it assumed a fatal character.

I ought to add, that I had not the charge of the poor woman's case above related, but saw her at the instance of a respectable practitioner in her neighbourhood, who had attended her husband, and assiduously employed all the known means of arresting inflammation." 161.

This ought rather to be termed an example of erysipelatous inflammation, than of genuine erysipelas. The distinctive character of this disease, is the abrupt border to the redness, not to mention the phenomena of its progress and results. We see no evidence of the existence of such characters in the case above related. Perhaps the most appropriate designation would be, diffuse inflammation of the throat.

We recollect two marked examples of this affection.

*Case* 1. A woman had compound fracture of the patella. Suppuration in the knee-joint followed. Soon after this, an erythematous blush appeared near one elbow, and pus was distinguished in the subcutaneous cellular tissue ; it was a purulent deposite. She went on thus for a few days, when she complained of great soreness of the throat. A general redness was ob-

* " The husband died suffocated, and I had been called to perform bronchotomy, but arrived too late ; he had expired."

served on the soft palate, from which it extended to the pharynx. The difficulty of swallowing augmented to a great degree, and difficulty of breathing followed. In this state, the poor woman died rather suddenly. The throat was not examined after death.

*Case* 2. A gentleman, while intoxicated, was thrown from his horse, and received a lacerated wound of both legs, and a simple, but severe fracture of one tibia into the knee-joint. Erysipelas of both limbs followed, and incisions were required to evacuate much matter, in the subcutaneous cellular membrane. The patient was very low and exhausted, and the erysipelas was still spreading up the thigh, when he suddenly complained of extreme sore throat. Only a general redness could be seen. In twenty-four hours after this he died, the dysphagia being complete. On examination of the throat, a diffused redness was found to be spread over the pharynx and palate, and to enter the laryngeal cavity. There was some, but not much œdematous effusion in the submucous tissue of the pharynx and the larynx.

Mr. Travers occupies eleven pages with remarks on gangrenous inflammation. Yet we do not perceive any necessity for noticing them, as their principal object appears to be, to point out a distinction between gangrenous inflammation and the death of a part. Mr. Travers seems to think that this distinction is not generally recognized. We conceive, however, that most well-informed surgeons do not hesitate to acknowledge it in theory, and, indeed, in practice. The popular, and when we say popular we mean the prevalent notion, even in the minds of medical men, is vague enough, if not erroneous, and the term mortification is indefinitely applied to sloughing from erysipelas, to sloughing from diffuse inflammation of the cellular tissue, to mortification from deficient circulation, from cold, and so on. The only mode of obviating this confusion is, to study the individual affections carefully. To illustrate this remark; we will take the first case related by our author.

*Case* 2. Mrs. —, æt. 77, pricked the inner side of her thumb with a thorn, on the 13th of July, 1833. She was of a full habit of body, and had "bad flesh to heal," and had suffered, in addition, from anxiety of mind.

The thumb soon swelled, and became painful. On the third day a surgeon was consulted. The injured part presented a slight elevation, much inflamed; the thumb and wrist were somewhat swollen. The pulse was frequent and full, and there were feelings of restlessness, general discomfort, and irritability of temper. The part was opened by a lancet, when a very small quantity of pus escaped, along with the minute point of a thorn. The pain and swelling increased daily. In about a week from the time of the accident, the cuticle began to separate in the neighbourhood of the injury, and the cutis beneath poured out a large quantity of lymph, which coagulated into a thick gelatinous crust. The vesication soon extended over the thumb, palm, and back of the hand, and now the cutis, which looked like buff leather, presented at two or three points, ulcerated openings, through which the cellular tissue might be seen in a sloughy condition. A free division of the cutis was made in several directions:- from the openings a little pus oozed out. Although the whole cellular substance was one mass of slough, yet the cutis remained sound, was sensitive, and bled rather profusely when divided.

The pain now diminished, but the health became more deeply affected, and low delirium supervened. Each day brought an extension of the vesication and subcutaneous sloughing, which now involved the whole thumb and hand, part of the fingers, and the wrist: and the forearm was greatly swelled and inflamed. The extension of the sloughing was followed up by fresh incisions through the cutis. Invigorating constitutional treatment was employed, but only with temporary benefit. The sloughs separated from the parts near the original injury, leaving a dissection of the muscles and vessels of the thumb; in the rest of the wound there was a more healthy suppurative process, and the sloughing made no progress up the fore-arm. On the morning of the 9th of August, however, the twenty-seventh day after the injury, there was a sudden failure of the patient's powers, and about noon she expired.

This case is denominated an example of gangrenous inflammation. A close analysis of its characters shews that it was one of diffuse inflammation of the subcutaneous cellular tissue. That tissue being inflamed, pus was deposited in it, and it sloughed, as it generally does. The sloughing of the skin was, as usual, secondary; it died, in consequence of the death of the cellular texture beneath it. Now surely it is better to term this affection what it was—diffuse inflammation of the subcutaneous cellular tissue, terminating in sloughing of the latter and of the skin, than to give it the vague name of gangrenous inflammation. For observe that, in the one case, the name definitively expresses the facts—whilst, in the other, the facts are not implied, though an accidental circumstance, the termination of the inflammation in sloughing, is. We say that this is an accidental circumstance, what logicians call an accidental mode. For the cellular tissue might not have sloughed, and then the term would have been inapplicable. It may be said that this is precisely the point, that the sloughing it is which renders the inflammation gangrenous. The answer is specious, but deceptive. In nine cases out of ten, nay, in ninety-nine cases out of the hundred, of diffuse inflammation of the cellular tissue, that tissue sloughs, unless incisions are made into it early; if incisions *are* made, the sloughing is arrested, and in parts where it has not yet occurred is prevented. Here, then, we perceive that the inflammation is or is not gangrenous, just as an accidental circumstance (the employment of the knife) is present or withheld. Yet the mode and character of the inflammation must be the same in either instance. We, therefore, object to this kind of nomenclature as incorrect, and, by consequence, unphilosophical.

Several other cases, resembling in many respects the preceding, are related by our author. We may pass them by, as they present no features requiring particular remark. The following are not devoid of interest.

Case 3. *Gangrene and Gangrenous Inflammation, from Ligature of the Femoral Artery for Popliteal Aneurism.*

A country labourer, æt. 45, had the femoral artery tied for popliteal aneurism. On opening the sheath of the artery, profuse hæmorrhage occurred, and, as the bleeding proceeded from the upper part of the incision, the opening of the sheath was extended, and the ligature placed higher upon the artery, when the bleeding and the pulsation of the tumor ceased. A second ligature was placed below the first, and the artery divided between the two: the external wound was then closed, and the patient replaced in bed. The foot shortly became cold; hot bottles were immediately applied to the limb. At 7, p.m. the foot was still cold, and pain was felt in it and in the leg. On the 6th, the leg and foot were still cold, and about the internal malleolus was a dark-blue streak. In the evening, the blueness was increasing in all directions, and sensation was lost in many parts. 7th. The blueness increased, and is mixed with yellow: hard, bright, semi-transparent surface of the foot; all beneath the skin seems adherent to it; complete loss of sensation of the foot and lower part of the leg. Excessive pain about the calf of the leg, excited by the slightest touch. Friction, and the carbonate of ammonia, were the means employed. On the next day, the discoloration extended to within three inches of the head of the fibula, and was bounded by an indistinct white line. This white line became more distinct, and the leg below it grew darker. On the 10th, there were vesicles upon the leg. On the 15th, this was black, much swollen, and tender, with several vesications. On the 18th, amputation by the circular incision was performed above the knee. The patient ultimately did well.

This would seem to be, and we have given it as, an instance of mortification from imperfect circulation in the limb. We see no great evidence of the existence, in it, of gangrenous inflammation. At all events, it is clear that the process, whatever we may term it, was widely different from that which occurred in the case of the lady after the prick of a thorn.

The next case is a curious one. We shall give it in Mr. Travers' words, as it is short, and insusceptible of useful abbreviation.

Case 4.—*Gangrene and Suppuration of the Hand and Fore-arm.*

"Sarah Ward, æt. 56; mother of a large family, long accustomed to drink freely of spirits and porter, with deficient animal, and, indeed, nutritious food of any kind, admitted 5th April, 1833. Three weeks ago, the summits of the fingers of the left hand became painful and swelled, with a sensation of pricking. The extremity, from the tips of the fingers to the shoulder-joint, is now swelled and variously colored, from a bright crimson to a dark blue; the disease bounded superiorly by a florid and exquisitely painful circle, the limb colder to the touch than natural, and exceedingly painful; the power of moving the fingers is lost, but they are not deprived of sensation. There is constant delirium, with high constitutional excitement; the pain confined to the limb. Half a pint of proof spirit, with a pint of the decoction of poppy-heads, to be applied warm every four hours, and a mild calomel and antimony pill to be taken night and morning.

10th. The same; to omit the pill, and take an opiate draught nightly.—Liq. ammon. acet. c̄ Vin. antim. 6 hor.

14th. Pain continues; delirium the same.—Ammon. carbon. c̄ Trâ hyoscy. ex infus. serpentariæ.

These symptoms continued for about two months after her admission, the pain gradually diminishing, and then leaving her slowly, from above downwards; the appearance of the red circle above-mentioned descended, and the part above it resumed its natural and healthy character. The loss of sensation in the hand and lower half of the fore-arm, has been complete from a fortnight after her admission.

May. There is a circle of inflammation about four inches below the elbow, and immediately below this is a line of ulceration, i. e. separation ; the hand and arm below are dry, black, hard, and shrivelled.

July. In this state she remained, with occasionally a sense of darting pain at the organized extremity; the denuded tendons were divided from time to time at the ulcerated part, and nitric acid lotion applied.

October. The bone was sawn through, and a portion remains to be thrown off, around which the integument is adherent, so as to form a puckered stump. The brachial artery is quite natural in its pulsation and volume, as is the arm in its general aspect." 191.

A case is given of gangrene of the left hand and arm. The patient was a very infirm old man. The arm was of a dark purple colour, cold and insensible, and emitted a very disagreeable fetor; in one or two places, the cuticle had peeled off. There was no distinct line of separation. The patient died. On dissection, there was found general ossification of the arteries and valves of the heart, particularly of the left subclavian artery.

Mr. Travers mentions the particulars of a case of gangrena senilis. An instance of sloughing of the fauces is more interesting. It was communicated to our author by Dr. Paris. The patient was a stout man, about the age of 50.

"On meeting Mr. P. professionally, as far as I recollect, about two months or more before his death, he asked me to look at his throat, which he said felt stiff and uncomfortable whenever he swallowed his saliva, and yet did not feel like an ordinary sore throat Upon examination, I could not discover any thing very remarkable, except that the tonsils and palate presented a darker hue than usual. He complained, however, of general prostration, and his pulse was feeble. I advised him to use a stimulating gargle, and after having opened his bowels, to take the sulphate of quinine. I do not, however, believe that he followed the advice with regularity; but he said he was better, and went to Ramsgate, from which place he returned and sent for me. I do not know the exact interval that had passed, but I should think about a fortnight. On my visit I found him still complaining of his throat, which had so much annoyed him, that he had, at his own suggestion, twelve leeches applied. I reprobated this practice, for I could see nothing more than a discoloration of the fauces, which had greatly increased since my last inspection. My plan was to support the powers of life, for which purpose quinine and a moderate quantity of wine were prescribed : the throat became more painful, and he could not put any of the muscles of the neck into action without much distress. As the disease advanced, he was unable to lower the jaw, so that all inspection was out of our power ; the tongue became covered with dark matter; deafness came on, his memory failed him ; the powers of life were evidently failing, and petechiæ appeared on the breast and legs ; the respiration was laborious, and his constant exertion to expectorate from the throat was highly distressing. On introducing the end of a tea-spoon between the teeth, we could perceive a very large and loose slough, which Mr. M.—— repeatedly attempted to remove by the forceps, but without success. On the night of his death, he brought up, by coughing, a considerable quantity of offensive dark-colored matter, evidently portions of slough ; his respiration became more difficult ; a cold clammy sweat covered the body, the pulse failed and he died.

As far as I can recollect, I should say that, from the commencement of his indisposition to his death, an interval of two months occurred, one half of which he spent as a decided invalid, never quitting the house.

I believe I have mentioned all the leading features of this curious case ; prostration of body and mind may be said to have constituted its most prominent peculiarity, without, in the first instance, any corresponding local affection in the throat'; that is to say, any local source of irritation in any way commensurate with the constitutional disturbance." 197.

Mr. Travers concludes the chapter by a few words on the treatment of gangrenous inflammation. We here see exemplified the bad effects of a loose phraseology. We have already shown that, under the denomination of gangrenous inflammation, several very different affections are included, and the same plan of treatment cannot be applicable to them all. This is a common yet a pregnant instance of the evils to which a loose nomenclature leads. The answer to the poetical inquiry—"What's in a name?" is obvious. In medicine a great deal. The following general observations of our author appear to us to be highly judicious, and should be borne in mind by all practitioners.

"A mistake often committed by practitioners is an immediate recourse to stimulants: great additional mischief sometimes results from it. I have often seen patients restored under a gentle course of carminative aperients, with occasional salines, and a diaphoretic opiate at night, where severe sloughing had already taken place; the inflammation of the surrounding part has abated, and the progress of the destruction has been arrested, and then tonics and wine have been administered with the happiest effect. Sometimes the tonic has been premature, and the patient has suffered a fresh access of inflammation, and been compelled to fall back upon the antiphlogistic for a longer time. On the contrary, after a dose or two of castor oil, I have in other cases resorted to the tonic and wine with equal advantage. As regards diet, good nutritious broths and jellies, or even a moderate allowance of solids may be given as soon as there is appetite for them, and I need not add that, in such circumstances, diet should take precedence of medicine." 200.

There cannot be a doubt that in diffuse inflammation of the cellular tissue leading to sloughing of it—in erysipelas terminating in mortification of the skin or of the subcutaneous cellular membrane—and even in the more legitimate case of gangrene and of sphacelus, the use of stimulants and tonics is abused, and bark and wine and brandy are poured in, with too little previous attention to the restoration of the secretions.

The following are Mr. Travers' sentiments on bleeding:—

"In the presence of acute pain or in full habits with flushed countenance, I have bled once, and in the onset of gangrenous inflammation generally with advantage. I have also bled where the inflammation has been established and extending, and where the immediate margin of the gangrene (not arising from cold or chemical disorganization) has been of an intense red color; but seldom, I must admit, with any obvious benefit or encouragement to a repetition." 201.

Here we see the difficulty of determining the exact cases in which Mr. Travers tried venesection. It would appear that there are cases in which this remedy is beneficial, although it must be owned that practical men enjoy few opportunities of seeing such. Though the loss of blood may be sometimes beneficial in the erysipelas and diffuse cellular inflammation that terminate, or tend to terminate in gangrene, yet the remedy should be used with discretion, and with caution. In the mortification resulting from cold or from defective circulation, we fancy that the lancet can be seldom dreamt of.

Local depletion is highly spoken of by Mr. Travers. Much must depend on the circumstances of the individual case, and on the judgment of the surgeon. The local depletion that often suits best is that which results from the employment of incisions.

In the general treatment, our author speaks highly, as most practical men have done, of opium. Attention to the secretions, and the proper exhibition of opium and of stimulants, may be said to constitute the essential principles of treatment.

We have now arrived at the termination of the first part of the present volume, excepting a supplement to the preceding chapters. That supplement contains what all must admit to be an eloquent defence of our author's views on the subject of irritation. From its very nature it is insusceptible of analysis, and ill adapted for criticism. We refer the candid reader to the work itself, if he wishes to see our author earnest in defending the opinions that he entertains. It is impossible for any one, possessed of the slightest portion of good feeling, to deny that, right or wrong, Mr. Travers takes high ground, and aims, if even he fails in arriving, at a high and intellectual course of study. With this general eulogy, which is not mere compliment, we enter on the consideration of the Second Part of the present volume.

This consists of five chapters. We shall not be enabled to complete the account of the whole in this article, but a short one in our next number will terminate the view of the matter which appears to us to be adapted for criticism or susceptible of analysis.

The first chapter treats—of the Nervous System and its Pathology—Sensation and Volition—Sympathy—Morbid Sensation—Morbid Action. For the opinions of our author on the various anatomical and physiological questions involved in the subjects here enumerated we must refer our readers to the thirty-seven pages of which the chapter is composed. Two sentiments, and two only, we shall quote.

"The physiology of the nervous system," says Mr. Travers, "is at this moment in a state the most unsettled, and perplexed by conflicts both of anatomical detail, experimental result, and theoretical opinion. The mere record of these differences would fill a volume." 227.

And "Experiment is extremely difficult of application, pregnant with fallacy; and closer examination convinces us that we are not yet in a condition to explain with accuracy the functions of the brain, as regards its several parts, more than the actual nature of those functions." 231.

That experiments have done the greater part of what has been done in the advancement of our knowledge of the phenomena and laws of the nervous system, cannot, we imagine, be denied. But, as might be anticipated, an extreme race of experimenters have arisen, who have mangled living animals in every conceivable mode and degree, and supposed they could determine the laws of life by this wholesale species of butchery. Those gentlemen who have sliced the brain in all directions, and determined each function by each slice: who have ascertained, by the edge of the scalpel, that one part impels us irresistibly forward, another backward, while another directs us to revolve upon our axis: those gentlemen, we say, who have been betrayed by experiments into such absurdities as these, have been useful in their way, by proving that what seem the most exact foundations for reasoning, may, by blundering heads and in blundering hands, be made the means of leading to the wildest and most ridiculous extravagances.

There is a point beyond which experiments cannot usefully be carried, on account of the disturbing circumstances that interfere with their application and exactness. The man of judgment is he who can determine that point most closely, who will give to experiments their real value, and will give no more. Beyond that point we must seek in the observation of functions, and in their alterations from disease, in the study of facts, in short, and in analogy, the collateral information which can no longer be obtained directly from experiments. With these remarks we proceed to the consideration of the second chapter.

### On Nervous Affections consequent upon Local Injury or Irritation, as Examples of Reflected Irritation, Morbid Affections of the Sensitive Nerves, Hysteria, Neuralgia, Morbid Affections of the Motive Nerves, Spasm, Tetanus. Cases.

The following passage on the subject of hysteria is not unworthy of extraction. It embodies many of its very common and very important phenomena.

"The condition of system termed in females 'hysteria,' exists, under certain modifications, in the male sex. It is a morbid condition of the nervous system, most frequently induced by the artificial restrictions which society and custom impose on the generative functions, but at all events, by the predominant influence which these exercise over the general system. Sympathetic in its nature and origin with the derangement of this important organism, it is witnessed principally at the period which marks its evolution and early development, but may exist at any point of the interval from thence to its decay.

The superaddition of this complex and mysterious mechanism to the simpler one upon which the preservation of individual life depends, is, under the negation of natural instinct, the prolific source of malady in the female, vague, various, and anomalous, beyond the compass of description. It is in the nervous system that its effects are displayed. Hence the digestive powers, the circulation, and all its subordinate functions, are troubled or interrupted, and the external senses, the temper, and moral character of the individual, even the faculties of the mind, are subject to be suspended, perverted, and impaired. The sensations especially are preternatural and morbid; and acute pain is referred to parts which may or may not have been the seat of injury, but which discover no vestige of inflammation or altered structure. In other cases, inflammation has run its course and left some slight traces of its existence, but none sufficient to furnish any satisfactory explanation of the suffering complained of. One very striking characteristic is the remittent and intermittent form of such pain, and the prevailing tendency to spasm; and with more or less caprice of appetite, irregularity of the secreting organs, and the habitual lassitude and exhaustion of the system, the absence of fever, and the maintenance of what is termed condition. Sleep too, though uncertain and of short continuance, is generally obtained in sufficient proportion. Imagination is troublesomely alive and active, and hence the exaggerated descriptions, the reveries, phantoms, noises, dreams, bizarre similitudes, and odd conceptions of patients so affected.

How often have we known patients treated for supposed diseases of the lungs, heart, liver, who persisted in referring acute pain to one or other of these regions, aggravated on pressure, in whom evidence of generally impaired functions was apparently confirmed by the coincidence of certain symptoms commonly present in organic affections, and confronted at the same time by the palpable signs of the hysteric aspect and temperament, the habitual leucorrhœa, the suspended, scanty, or redundant menstruation, globus, pale urine, &c. &c. and especially the long duration of the disease without wasting." 268.

Within these last few years attention has been directed to many nervous affections in the female, which seem dependent on the state that, for want of a better name, we designate hysterical. Thus pains are experienced by young females in the joints, especially in the articulation of the knee, which have been and which are very commonly mistaken for serious disease, but which are in reality merely nervous. Pains are felt in the chest and abdomen, counterfeiting pleurisy, hepatitis, peritonitis, and other inflammatory maladies. Perhaps the leading feature of distinction between the genuine phlegmasiæ and these bastard hysterical complaints—is the disproportion between the pain and the attendant symptoms. In the hysterical affection of the knee, for example, although the suffering seems to be excessive there is very little swelling, and the progress of disorganization is not what it must have been, if serious disease had existed. Exaggerated pain, then, and comparatively insignificant general symptoms, or symptoms clearly marking the hysterical constitution, are the leading characters of local hysterical affections. They are so frequent as to render an accurate acquaintance with them indispensable to every practical man, who would desire to avoid the commission of daily and not inconsiderable blunders.

As Mr. Travers equally judiciously and feelingly observes, it is quite erroneous to suppose that such complaints are hypochondriacal, or in any degree simulated; there are none in which the patient is more willing to submit to severe and even deforming modes of counter-irritation, and none in which these more completely fail of giving relief. But though the patient is not conscious that the pain is mental, in one sense it undoubtedly is so, or rather it depends on a general state of system which exaggerates and vitiates the ordinary sensations.

In proceeding to discuss the subject of neuralgia, Mr. Travers points out analogies between it and chronic rheumatism, analogies, however, which we cannot think altogether unexceptionable. He observes, for example, that quinine and steel and arsenic and the volatile tincture of guaiacum and the cold bath are among the best remedies for chronic rheumatism. We really were not aware that the cold bath was a good remedy for chronic rheumatism, and we should feel extremely loth to prescribe it for a case of that complaint. Hot and vapour baths are ordered frequently enough, but cold baths, we suspect, are but charingly administered. But Mr. Travers, as we said, maintains the analogy between neuralgia and chronic rheumatism, and in its widest sense he explains it thus:—

"But as irregular circulation may depend upon many causes besides inflammation, and as there can be no doubt from the continual proofs which are exhibited to us, that the capillary circulation is especially influenced by the nervous fibre, and since as often before observed, irritation and inflammation are essentially distinct, it is not too much to infer that it is on the side of the nerves that the balance of action is suspended primarily, in cases presenting no symptom of inflammation throughout their course, if we except pain; and secondarily, in a certain proportion of bygone cases of actual inflammation, in which pain alone remains after all other signs of inflammation have disappeared. It is on this principle that I explain the sudden attacks of tic douloureux after exposure to the cold produced by evaporation—of sciatica after sitting on damp ground—of irritated nerve by an old unsound tooth, or a portion of dead bone, or any foreign body acting upon a nerve of sensation, as the ligature including a twig after amputation and its confinement by cicatrization, whence the pain of stumps long healed;—and thus that I reconcile the points of analogy between the phenomena of chronic rheumatism and neuralgia in its largest sense, concluding that the morbid sensation, like the natural, varies according to the texture in which the nerve is distributed, and that the neuralgia may be and often is a secondary as well as an idiopathic or primary affection; and lastly, thus that I explain the efficacy and the success of similar remedies and modes of treatment in these different diseases." 280.

This explanation may carry more clearness with it to our readers than it does to ourselves. We unwillingly confess that it does not render our ideas upon the subject much more definite than they were before. Whatever the analogy may be, the distinctions between neuralgia and chronic rheumatism are obvious. The latter is clearly an inflammatory process, displaying during its progress the characters of inflammation, and exhibiting its sequelæ. The former neither presents the signs of distinct inflammatory action present, nor the traces of it past. And although it is true that general tonics agree with chronic rheumatism when very chronic, it is true also that local antiphlogistic treatment is equally if not more beneficial. After all, the question is a very idle one, for we know so little of the real nature of either neuralgia or chronic rheumatism, that the explanation of one by the other, is little more than the attempt at defining one unknown quantity by comparing it to another equally uncertain.

Morbid affections of the motive nerves are next passed in review by Mr. Travers. If one set of muscles are paralysed, their antagonists prevail. Of this we see daily examples, and to it Mr. Travers alludes. He alludes also to some other kinds of paralysis.

"A modification of paralysis, sometimes congenital, sometimes occurring in infancy from dentition and other causes, is a deficiency of control, and consequently a want of consent in the actions of muscles. These are the cases in which the child totters and is in danger of falling if capable of supporting himself, and the grown person with difficulty maintains his equilibrium, and in order to do so enlarges his base, performing all the motions of progression circuitously or by jerks. In these cases, which are depending upon an originally checked or imperfect development, the entire voluntary muscular system is affected, so that stammering interrupts the speech as much as sudden halts the gait. In extreme age the loss of command approaches almost to the same state, but in this, permanent tremor or shaking palsy is more frequent; another modification of impaired nervous tone. That a condition somewhat similar may depend on the state of the cerebral circulation, is seen in the delirium tremens or tremulous excitation of drunkards and debauchees; it is also from the same cause an occasional and temporary consequence of severe nervous ailments and brain fevers. Overstrained and painful extension of the arm or leg will induce the same loss of command as regards their muscles, and to a certain extent, the undue interference of the mind with the effort. All these cases are arising from a deficient or impaired innervation, and are to be regarded as of a passive character." 281.

Mr. Travers, after speaking of chorea, adverts to a form of affection of the motive nerves, the nature of which is doubtful, and the character extremely obstinate. In it, the muscles are subject to be thrown into irregular and involuntary action by causes not obvious, and which must be supposed to depend on some mechanical irritation of their motive nerves, probably an unequal compression, from the encroachment of some bony growth upon the spinal theca, or the orifices by which they make their exit, or from contiguous bone in some part of their course, or a deposition in or upon their sheaths; it is generally but not always a painful disease. In this case, the sensitive being affected as well as the motive fasciculus, renders the first suggested hypothesis the most probable. The affection is more or less extensive, peculiar to a limb, or affecting a part of the body, or even the whole trunk. It is sometimes incessant, rendering artificial support necessary to maintain even a precarious and deformed position, or accomplish progression in any sort; sometimes it occurs only in paroxysms at uncertain intervals, or in such efforts as require and cannot be performed without the action of the affected muscles.

This chronic convulsion is little if at all remediable. Mr. Travers attended, with the late Dr. Babington, a lady who had been afflicted thus for years, in the muscles of the nucha and left side of the cervical spine. No organic cause could be detected. The disease was not benefited by various modes of treatment, nor was the pain relieved by any narcotic or external application.

"There is a form (continues Mr. Travers) of the maniacal paroxysm in which the hapless lunatic will sing the same tune, utter the same cry, or repeat the same words, and perform precisely the same evolution or contortion of the muscles for days and nights together and suffer no interruption, with such accuracy and regularity, that the living machine seems converted into an automaton. Without any affection of the mind, one sometimes sees cases of involuntary motions of the muscles of the limbs or trunk, equally symmetrical in their order and extent, continued and resistless. Most extraordinary but well-authenticated cases of this kind are on record. We must refer these attacks to some temporary morbid condition of the brain and spine, without which they could neither be set agoing nor maintained. If superadded to organic change, they are not simply enduring but permanent; not intermittent, but subject to frequent paroxysm, and soon destructive from exhaustion and interruption of important functions." 284.

Epilepsy is next noticed by our author. When "diffuse epileptic convulsion" succeeds an injury of the head, it is almost always speedily fatal. Convulsion on one side and paralysis upon the other, as well as total paralysis, Mr. Travers has seen recovered from, after injuries of the head. As a symptom of apoplectic effusion in the brain, it has been, in Mr. Travers' observation, generally fatal. Mr. Travers mentions an interesting case of symptomatic epilepsy, which occurred many years ago at St. Thomas's Hospital, under the care of Mr. Birch. A boy who was admitted for these fits was discovered to have a pit or depression of the skull, upon which pressure created uneasiness. Upon that spot the trephine was applied, and the concave piece removed; at the instant of turning it up he had a sharp epileptic fit, and this was the last. From the internal table a spiculum, a quarter of an inch long, projected, pressing upon the dura mater. The operation threw as much light as the morbid growth upon the cause of the fits, the occasional state of the circulation rendering the membrane liable to the same offence as it received from the slight pressure or irritation occasioned in turning up the loosened piece.

As our author observes, a plethoric and exsangueous state are both conducive to the state of convulsion. It is not uncommon for persons to be affected with partial paralysis after great loss of blood for convulsive affections, symptomatic of disordered stomach. A dyspeptic gentleman who complained of pain in the head, with a slight degree of stupor, or rather slowness of intellect, and lost blood immediately by the advice of his physician, found that one side of his face was paralyzed after the operation, from which state it has slowly and imperfectly recovered, though it happened two years ago.

Spasm of the involuntary is, fortunately, less frequent than that of the voluntary system of muscles. Yet a certain amount of spasm of the former is not very uncommon.

"When the muscular structure becomes inflamed, as in acute rheumatism of the heart, or the venous side being over-loaded it is incapable of relieving the pulmonary circulation, it becomes subject to spasm, which terminates life suddenly. When the supply of its nutrient blood fails from the constriction of the mouths or ossification of the walls of the coronary arteries, it is also subject to fatal spasm under any excitement which induces its increased action. The spasm of the pylorus, and of the gall-duct, and of the intestine in its course, are predisposed to by irritation of the villous surface, and is also a symptom of obstruction and inflammation. The pain attending these affections recurring at short intervals, is most acute, especially the spasm of the ureter, of the sphincter ani, and of the neck of the bladder in dysuria, whether from irritation or inflammation.

Spasm of the cremaster muscle is rare, but I have seen it so severe as to make the patient scream with the agony, the testes being perfectly sound, as well as when inflamed.

In all the cases we have referred to, irritation is a competent and frequent cause of spasm, which is a temporary, irregular, and involuntary action of muscles, attended with pain and followed by exhaustion, and often preceded by a notable or discoverable cause of irritation of the nerves supplying the part affected, either at their origin, in their course, or at their termination. The contractions produced in a fresh-killed animal by galvanism convey the best idea of the connexion subsisting between the nerve and muscle, and the effect of irritation acting directly upon the muscle, or upon the muscle through the nerve." 289.

The following is a summary of the facts to which we have partially drawn the attention of our readers.

"We have seen that the nerves of sensation and motion are subject to varieties of paralysis from checked or impaired development and extreme age, as well as from disease incident to all periods of life. It may affect exclusively sense or motion, or include both. It may be partial, limited to one side, one limb, one district, and set of muscles, a single muscle, the point of a finger, or affecting the whole system. It may be a direct or an indirect result of injury or irritation, and the injury or irritation may be adjacent or remote. But as sensation and voluntary motion are principles, resident not in the parts, but in the brain and spinal chord, the deficiency must in either case depend on some interruption to the transmission, or some deviation in the supply of these faculties. Whether the cause of irritation exists in the organ of external sense or the brain, the stomach or the limb, the morbid alteration of sensation and motion is only to be explained by an alteration, organic or functional, in the brain supplying or nerve conveying the principles of sensation and motion. Hence if we amputate a limb, the man is troubled with the delusive sensation and motion of that hand or foot of which he is bereft from irritation of the remaining nerves, the instruments which conveyed sensation and motion, and the brain, the organ by which they were supplied. What determines the various modes and seats of paralysis of sensation or motion in cases of organic change, we have sometimes opportunities of seeing either during life or at its close; but in a large proportion of cases we see the symptom only, the cause is hidden from us. Still more is this the case with the affections of irregular muscular action, since a yet larger number of these are sympathetic, *i. e.* due to temporary circumstances, such as disordered circulation or innervation, and leave no trace behind." 290.

### Tetanus.

The consideration of tetanus forms the conclusion of the chapter. We know not if Mr. Travers will throw much light upon this disease—as obscure in its pathology as uncontrollable in its progress. Mr. Travers, indeed, offers no systematic description of its phenomena, and wisely avoids that more than twice-told tale.

Mr. Travers, regarding the malady through the medium of his views on direct and reflected irritation, seems to think it a mistake to consider tetanus as divisible into idiopathic and traumatic. For, says he, whether the morbid contents of the intestinal canal, or the effects of a sudden chill of the surface, or an exhaustion of nervous power, or the irritation of an external injury, prove exciting causes of the tetanic spasm, the disease must be equally regarded as symptomatic, and the predisposition as the circumstance which explains the varieties of susceptibility. The same reasoning would equally apply to the case of most other diseases. Erysipelas, for instance, or pleuro-pneumonia, may occur idiopathically, or as a consequence of injury. By the division into idiopathic and traumatic, it is not meant to say that no local cause exists for the former, or that the mere injury constitutes the whole and sole cause of the latter. It is simply a rough and convenient nomenclature—founded on a very obvious distinction—and useful, because the two forms of the complaint are generally observed to display some differences. Traumatic erysipelas is usually more severe than the idiopathic form, and has a greater disposition to affect the cellular tissue ; and traumatic tetanus is confessedly more violent than its idiopathic brother.

The period of accession of tetanus, after injury, varies from an hour to ten days or a fortnight. In some rare instances it is almost immediate. The case of a negro is recorded by Dr. Robison, in which the spasms commenced in a quarter of an hour after the infliction of a puncture with a fragment of china-ware: and some years ago a man was brought into St. Thomas's Hospital for a recent fracture, in a state of universal tetanus of tremendous violence, which proved fatal in a few hours. The fracture was an oblique one of the thigh-bone, which, penetrating the rectus muscle, was continually playing through its belly in a see-saw. Mr. Travers has seen the disease established, under similar circumstances, on the fourth day ; the fractured portion had penetrated the vastus internus. This case was in Guy's Hospital, and proved fatal on the seventh day. The interval preceding the attack does not determine the form of the disease, *i. e.* the disease is sometimes of the severest and most rapid description where an interval of ten days has elapsed. The late unfortunate Earl of D—— was seized on the fifth day, from the accidental amputation of two of his toes by an axe. The disease was of the severest form and proved fatal on the seventh. On the other hand, where the disease follows close upon the injury, it is for the most part uncontrollably rapid and fatal.

The form of injury that occasions lock-jaw is subject to almost every imaginable variety, save one. Mr. Travers has never known a clean incised wound, as from the surgeon's knife, occasion it. Baron Larrey saw the disease produced by a fish-bone lodged in the throat, and two cases are mentioned by Mr. Morgan, following the blows inflicted by a schoolmaster with his cane, and both fatal. Even the injection of a hydrocele has been followed in the West Indies by locked-jaw ; and Mr. Travers has now under his observation chronic trismus, from the extraction of a tooth, with injury to the jaw. The most frequent seat of the injury is in the hand or foot.

"As we have said, the period of access varies, so must the stage at which the local injury has arrived; but in flesh wounds the period of commencing cicatrization, after the mundifying process is completed, seems to be most liable to the attack of spasm; not unfrequently the punctured wound, as from a nail, wears the aspect of being healed, and is almost forgotten when the spasms set in. These facts are now so established by repeated observation, that it is difficult to disconnect the phenomenon of the incipient spasm with the altered condition of nervous and muscular structure in the healing or newly-cicatrized part. Ligature of the funis umbilicalis, of the entire spermatic cord, and the anterior crural nerve, are among the known causes of tetanus. The former is common in hot climates; the two latter I have myself seen. Now cicatrization is adhesion and fastening of parts before free and moveable on each other, and approaches much to the nature of a ligature. In adverting to a mechanical condition, we of course admit all such varieties as may tend to the production of a similar effect, and we should expect that fastening a naturally free part would be equivalent to its strangulation or confinement by pressure of any kind, the interruption to its function being the same. Destruction of the part by caustics and its removal by amputation have been resorted to ineffectually when the symptoms have commenced, and I have twice known the disease ensue after amputation. But if, when the circumstances admit of it, wounds likely to produce tetanus, were treated while recent like those inflicted by a strange dog, so as to destroy, or at least alter the sympathies and mode of healing, it would probably be of more rare occurrence." 295.

We are disposed to agree with our author in his conjecture, that the alteration occasioned by contraction and consolidation of tissues, is operative in giving rise to the peculiar irritation that ends in tetanus. That it is not the sole cause is evident, from the variety of circumstances and conditions under which that disease originates; but still, of many causes, this appears a frequent one. We have seen one case and heard of another, (not to mention recorded instances), in which the puncture of a considerable nerve, and the subsequent formation of a sort of ganglion in it, by the deposition of coagulated lymph, gave rise to lock-jaw. In the case we witnessed, the internal plantar nerve had been punctured in climbing over an iron railing. On dissection after death, this nerve was found involved in a small hard cicatrix. In the case of which we heard, the peroneal nerve had been wounded by a pitchfork. It was similarly involved in a ganglionic cicatrix. Yet, in other instances, this occurs without the supervention of tetanus. We remember Sir Charles Bell's describing a case, in which the posterior tibial nerve became the seat of a sort of a ganglion, formed by the deposition of lymph, in consequence of a man falling from a height on a projecting substance that caught the calf of the leg. This individual suffered from the most agonizing pain in the course of the nerve affected. His tortures were such, that he gradually wasted, and ultimately died from their severity and continuance.

As Mr. Travers justly observes, the susceptibility to the tetanic spasm varies exceedingly. It is often seen that a wound or injury, so likely to produce the disease that we are daily on the watch for it, passes uninterruptedly through its stages; it is also seen by those who have the opportunity, that a menace or premonition of the attack is often decidedly exhibited, and yields to continued and active purging, or a free incision. Now and then, but not often, it takes the most experienced surgeon by surprise. In short we must confess, that though sometimes, as in the instance of implication of a large nerve, we think we can philosophically account for the supervention of tetanus, in the majority of cases we are unable to lay that unction to our soul, and our ignorance must be honestly admitted.

Mr. Travers, indeed, seems inclined to lean to the hypothesis that places the general cause of tetanus in the alimentary canal—a hypothesis slightly countenanced by a small number of circumstances, but not plausibly supported by any extensive induction. Yet practically, the notion is not devoid of value, because it induces those who might otherwise neglect them altogether, to attend to the alvine excretions ; and in chronic tetanus it is more than probable that such attention is necessary and useful.

In tetanus, says our author, who collects what has been observed in favour of its intestinal origin—in tetanus not only the most obstinate costiveness prevails, but when, by powerful medicines, the bowels are relieved, matters the most unhealthy and unnatural, as Mr. Abernethy observes, " quite unlike fæces," are voided. We suppose the scybalous condition to be an effect, as probably it is, of the disease, but how do we know that the morbid quality, quantity, consistence, &c. of the contents of the canal were not existing previously to the attack of spasm, and operating with all the force of a powerful local irritant upon the highly irritable nervous tissue of the canal. Abrasions, and small ulcers of the villous coat and mucous glands of the alimentary tube are seen continually in slitting open the canal, where they were wholly unsuspected; and he, Mr. Travers, knows no reason why, "when we find idiopathic tetanus and dysentery springing up under the same external conditions of the body, the local condition which is competent to the production of tetanus, from the analogy of the traumatic form, should not be so considered in that, of which the local cause is hidden during life, and, therefore, supposed to be simply a morbid condition of the general system." The latter sentence and the latter thought are almost equally obscure. It is a pity that words are possessed of the power of simulating definite ideas. Their ignis fatuus meaning too frequently betrays the author and the reader into their pursuit, as of a real light. If the sentence we have quoted admits of the reduction into any expression, it is surely this, and nothing more—that as cold is a cause of dysentery and of tetanus, cold may be regarded as a cause. We have hunted through the devious thicket of words, and, in their tangled meshes, we beat up no other game than this. But to continue.

Drs. Macarthur and Dickson have given dissections, shewing inflammatory conditions of the bowels, in four cases of acute tetanus. We want more precise and extended information on this subject. Certainly the traumatic tetanus of this country does not bear out the opinion of visceral inflammation. Instances of indigestible substances, and of tape and round worms found on inspection of the canal in tetanic cases, are on record ; and a case of trismus, under Mr. Earle, recovered after the expulsion of a tape-worm from the bowel.

We said that the theory which makes visceral affection the cause of tetanus will not bear examination, but still we repeat that in practice it should be remembered. Whether depraved secretions produce a disease or are its consequence, still per se depraved secretions are injurious. We proceed with the pathology.

In a considerable number of cases anomalous appearances of ossific deposition have been met with upon the arachnoid coat of the spinal chord, or upon the processes of the dura mater, and some unusual saliency of the ridges or processes of the cranium has been noticed in examinations post mortem. Such appearances, certainly, cannot be regarded as necessary causes of tetanus, yet probably they operate by predisposing to undue irritability of the nervous system.

In the following sentiments we quite coincide:—

"I should say that the evidence before the public to establish that tetanus has its origin in inflammation of nervous structure has failed, and that few if any practical surgeons of experience entertain such a notion. The spine, the ganglia, the nerves of the wounded part have been repeatedly and minutely inspected in the worst cases of tetanus and other spasmodic diseases, and no such appearance discovered. Even the common statement of increased vascularity and effusion beneath the investing membranes are as common in other acute diseases as in this, and these are by no means universal in tetanus. Very rare instances have occurred of a nerve being included in the injury, and palpably altered in consequence in common with other textures by inflammation; but even in such cases, I should, from reference to the whole phenomena, be inclined to question its operation as a cause, for wounds of nerves happen continually without betraying any tendency to the disease." 300.

We coincide in the former, but we do not coincide in the latter opinion. No doubt there are many local causes of tetanus, but we do not see why injuries of nerves should be arbitrarily excluded from the list. The evidence on their side is every way as good as that in favour of the rest.

The following facts are not unimportant. After stating that there is no evidence of the direct implication of either the vascular or absorbent system in tetanus, Mr. Travers remarks that:—

"It has happened that inflammation of the bronchia and lungs has suddenly supervened upon the subsidence of the spasm, and in the form of effusion proved fatal in a few hours. So in a case I have already given, tetanus supervened upon erysipelas, and in another yet to be mentioned, the same thing occurred. The danger of metastasis is greatest in all those diseases which are essentially nervous, or being inflammatory, are under the special influence of the nervous system, as e. g., aggravated hysteria, chlorosis, and convulsion of all kinds; erysipelas, gout, rheumatism, &c. From the plunge of the cold bath I have seen the tetanic patient brought up a corpse, and I have known an hydrophobic patient expire in the spasm induced by the act of excising the wound. In neither case was there any premonitory sign of impending dissolution: the symptoms were but of a few hours. A gentleman under the care of Mr. Key, who had just recovered from a severe attack of tetanus, was thrown into a fit of passion by some ill-timed communication relating to his property, and died on the spot, probably from spasm of the heart. In a chlorotic girl I have known the shock of a shower-bath fatal; and chorea sometimes terminates suddenly in hydrocephalus. The tendency of erysipelas to seize the head, and rheumatism the heart, and gout the stomach is well known.

The practical inferences are twofold which we may draw from these facts; the imperative necessity of procuring and preserving free and healthy secretions from the visceral glands, and of caution to avoid sudden shocks or surprises of the greatly debilitated system." 302.

We observed that the facts were not unimportant, but we cannot help remarking that in the passage we have quoted, there is a strange mélange of circumstance and theory, of premiss and conclusion that does not seem to follow on it. Thus chlorosis, erysipelas, and rheumatism are placed in the same category—that of diseases essentially nervous, or, though inflammatory, being under the special influence of the nervous system. We scarcely know why either erysipelas, gout, or rheumatism should be said to enjoy the special benefit of nervous superintendence. Nor, supposing all this be literally true, do we perceive the justice of ranking sudden death from cold affusion as an instance of metastasis. We should call it an example of the bad effects of a sudden shock upon the system, and although, if hard-pressed, we might find it difficult to define what a shock on the system is, or the exact modus of its action, we think we could still be enabled to prove that it does not correspond with what is usually held to be metastasis. Metastasis is the translation of some obvious action from one part to another more or less remote. What proof of such translation can our author offer in the sudden death in the shower-bath of a girl affected with chorea, or in the equally sudden demise of a patient labouring under tetanus, when submitted to the cold affusion?

On the matter of fact we would offer a word or two. We may grant that hysteria is essentially a nervous affection, and perhaps we may admit that chlorosis is so too. But we are not equally inclined to allow that, in these complaints there is a marked disposition to metastasis. In rheumatism and in gout we have the best examples of this phenomenon. But observe the circumstances in either instance. The same tissue, a diffused and pervading tissue is in both cases affected. In rheumatism this is true au pied de la lettre. The fibrous or the cellular tissue is affected in the foot, and the fibrous or the cellular tissue is affected, if the pericardium or the pleura is invaded.

In the propriety of the following general observations upon treatment we agree. They do not exclusively apply to tetanus.

"The nervous pathology is to be viewed first, as regards its proper system; secondly, as regards its associations with the vascular. In such affections of nerves as are peculiar and exclusive, we may conform our treatment to the first view; in those which are resulting from or mixed with the arterial and venous system, we must modify it in obedience to that connexion. Pain, depending on a morbid condition of nerve, is aggravated by such measures as allay pain proceeding from inflammation. The same may be said of spasm not depending on inflammation or mechanical obstruction, inducing or threatening inflammation. In the acute stage of inflammation we do not give tonics; but in the chronic stage, where the consequences which it has left constitute the disease, we administer them most beneficially. In the paroxysm of intermittents, we carefully withhold such tonic remedies as exhibited in the intervals of the paroxysm break the train, and prevent its accession by fortifying the nervous system. Functional disorders and irregularities are counteracted and cured by such agents as operate upon function, i. e. upon the brain and nerves; whether acting directly upon the trunk or the extremities of the arterial tree, upon the pulmonary or the aortic side, or not upon either directly, but intermediately." 302.

Here we must terminate this article. In our next, we shall dispatch the remainder of the work. It contains many facts, much reasoning, and a certain degree of speculation. Our habits and our conviction lead us to pay more attention to the former than the latter. There are some, we know, who look upon a disposition to relish mere facts, as evidence of an inferior kind of intellectual appetite. To those transcendentalists, we leave their piquant and unsubstantial viands, and, contenting ourselves with our homely but nutritious fare, we venture to expect a mental soundness which ragouts and sauces seldom give.

